# Integration bile acid metabolomics and gut microbiome to study the anti-liver fibrosis effects of total alkaloids of *Corydalis saxicola* Bunting

**DOI:** 10.1186/s13020-025-01158-2

**Published:** 2025-07-04

**Authors:** Qianyi Wang, MeiLing Zhang, Mingwei Meng, Zhuo Luo, Ziping Pan, Lijun Deng, Jinghua Qin, Bingjian Guo, Dan Zhu, Yanmin Zhang, Hongwei Guo, Yonghong Liang, Zhiheng Su

**Affiliations:** 1 Department of Pharmacy, Guangxi Medical University Cancer Hospital, Nanning, 530021 China; 2 Pharmaceutical College, Guangxi Medical University, Nanning, 530021 China; 3 The First People’s Hospital of Nanning, The Fifth Affiliated Hospital of Guangxi Medical University, Nanning, 530021 China; 4 School of Pharmacy, Health Science Center, Xian Jiao Tong University, Xian, 710061 China

**Keywords:** Total alkaloids of *Corydalis saxicola* Bunting, Liver fibrosis, Bile acid, Gut microbiota, Fecal microbiota transplantation, *Lactobacillus reuteri*

## Abstract

**Background:**

Bile acids and gut microbiota participate in the pathogenesis of liver fibrosis (LF). The total alkaloids of *Corydalis saxicola* Bunting (TACS) is a traditional Chinese medicine extract that has been used to treat LF, but the underlying mechanisms are not clear. This study performed integrated metabolomics and gut microbiome analysis to study the anti-LF mechanism of TACS using a rat model.

**Methods:**

Ultra-performance liquid chromatography quadrupole time-of-flight mass spectrometry (UPLC-Q-TOF/MS) was used to identify the chemical compounds in TACS. Biochemical and histopathological analysis were performed to determine the efficacy of TACS. Bile acid-targeted metabolomics was used to assess changes in the bile acid (BA) profiles in TACS-treated LF rats. 16S rRNA gene sequencing and metagenomics were used to assess changes in the gut microbiota of the TACS-treated LF rats. Antibiotic cocktail treatment and fecal microbiota transplantation (FMT) were used to determine the relationship between the gut microbiota and the anti-LF effects of TACS. Metagenomics was used to identify significantly enriched gut microbiota after TACS treatment and its correlation with the anti-LF effects was verified by in vivo experiments.

**Results:**

TACS treatment significantly reduced the levels of serum liver enzymes, fibrosis and pro-inflammatory cytokines in the liver. TACS significantly increased the levels of chenodeoxycholic acid (CDCA) and taurochenodeoxycholic acid (TCDCA) in the cecum and decreased the levels of cholic acid (CA) and deoxycholic acid (DCA) in the liver of the LF rats. TACS significantly increased the abundances of *Lactobacillus* and *Akkermansia* in the LF rats. Antibiotic cocktail treatment and FMT have shown that the effect of TACS cure liver fibrosis depends on the gut microbiota. The abundance of *Lactobacillus reuteri* was significantly increased by TACS. Administration of *Lactobacillus reuteri* via gavage ameliorated LF.

**Conclusions:**

TACS exerted anti-LF effects in rats by modulating bile acid metabolism and gut microbiome.

**Graphical Abstract:**

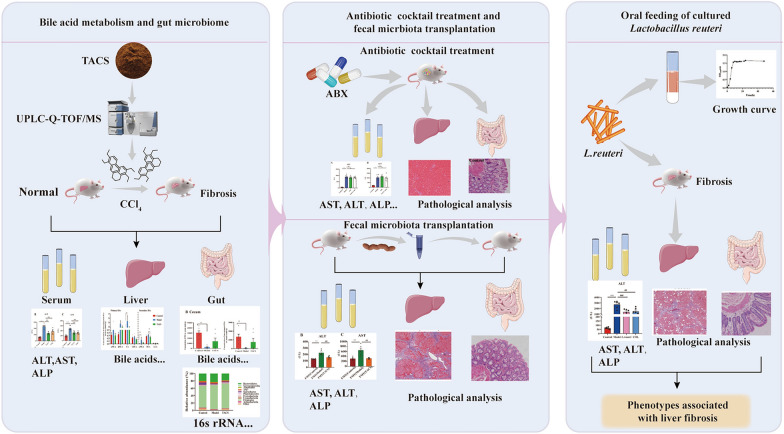

**Supplementary Information:**

The online version contains supplementary material available at 10.1186/s13020-025-01158-2.

## Introduction

Liver fibrosis (LF) is a common trauma healing chronic liver disease, which is characterized by excessive accumulation of extracellular matrix (ECM) and tissue scarring [1]. If LF is not treated effectively, it can develop into cirrhosis and hepatocellular carcinoma (HCC). Therefore, identification of pathological mechanisms involved in LF development is important for developing effective drugs for cure liver fibrosis.

Abnormal bile acid metabolism is closely associated with the pathogenesis of LF [2]. Bile acids (BAs) are important signaling molecules that drive the progression of LF by binding to receptors on various liver cells, including hepatocytes, macrophages, and hepatic stellate cells (HSC) [3]. Furthermore, primary BAs are synthesized in the liver and are converted into secondary BAs by the gut microbiota. Taurodeoxycholic acid (TDCA) and glycodeoxycholic acid (GDCA) activate hepatic stellate cells and significantly up-regulate the expression of fibrosis marker proteins [4]. Metabolomics based on mass spectrometry is an effective tool for evaluating the efficacy of traditional Chinese medicine (TCM) because it can be used to systematically determine changes in the metabolic profiles of biological systems under pathological conditions and identify clinically useful biomarkers [5].

Recent studies have demonstrated that gut microbiota dysbiosis plays a crucial role in the occurrence and development of liver fibrosis, whereas supplementation of probiotics alleviates LF [6, 7]. Gut microbes such as *Firmicutes*, *Bacteroidetes*, *Escherichia coli,* and *Akkermansia muciniphila* are associated with LF [8, 9]. Patients with LF demonstrate increased abundance of *Streptococcus* and *Clostridium* and reduced abundance of *Prevotella, Akkermansia*, *Enterococcus* and *Eubacterium* in the gut microbiome [10, 11]. Gut microbes also affect the development of LF by altering BA metabolism and signaling pathways [12, 13]. *Lactobacillus,* well-known BSH-producing bacteria, alleviates LF by modulating bile acid metabolism [7]. BAs are synthesized and secreted in the liver cells and carried into the intestine through the biliary tract. BAs influence the composition of gut microbiota and regulate the host enterohepatic circulation metabolic pathway and immune system through the farnesoid X receptor (FXR) [14, 15]. Therefore, precise profiling of the gut microbiota and BAs may provide new insights into the mechanisms through which they regulate liver fibrosis.

Total alkaloids of *Corydalis saxicola* Bunting (TACS) are the main active components in *Corydalis saxicola* Bunting and exhibit hepatoprotective and anti-bacterial effects [16]. Our previous work has revealed that TACS could mediated gut microbiota dysbiosis and bile acid metabolism pathway in antibiotics rats[17]. Moreover, TACS alleviates gut microbiota metabolism disorder in the liver injury and liver fibrosis model rats [18, 19]. However, the effects of TACS on the bile acid metabolism and gut microbiota in the LF model rats need to be characterized in detail.

This study combined bile acid-targeted metabolomics and gut microbiome to analyze the changes in the bile acid metabolism in the liver and the gut microbiota in the intestine after TACS treatment in the LF model rats. Furthermore, 16S rRNA gene sequencing analysis was used to analyze the status of the gut microbiota disorder after TACS treatment. Antibiotic cocktail treatment and FMT were used to investigate whether the anti-LF effects of TACS were dependent on the gut microbiota. Metagenomics was used to identify the bacteria that were significantly enriched by TACS. Subsequently, the anti-fibrosis effects of the bacteria were verified in LF model rats. These findings not only provide a novel mechanistic perspective for the TACS treatment in LF and a reference for developing probiotic drugs for the treatment of LF.

## Materials and methods

### Chemicals and reagents

Carbon tetrachloride (CCl_4_) solution, olive oil and colchicine were purchased from Adamas (Shanghai, China), Macklin (Shanghai, China) and Xishuangbanna Pharmaceutical Co., Ltd. (Jinghong, China), respectively. ELISA kits to estimate lipopolysaccharide (LPS), tumor necrosis factor-α (TNF-α), interleukin-6 (IL-6), and interleukin -1β (IL-1β) were purchased from Shanghai Pan Ke Industrial Co., Ltd (Shanghai, China). Vancomycin (H30J12Y138789, purity ≥ 98%), streptomycin (S06M11Y112378, purity ≥ 98%), ampicillin (A16GS157597, purity ≥ 98%), and gentamicin (J10GS153398, purity ≥ 98%) were purchased from Shanghai Yuanye Biotechnology Co., Ltd (Shanghai, China). De Man, Rogosa and Sharpe (MRS) medium was purchased from Haibo Biotechnology Co., Ltd(Qingdao, China).

### Preparation and qualitative analysis of TACS

The *Corydalis saxicola* Bunting herbs were obtained from Donglan County, Hechi City, China, and authenticated by Professor Dan Zhu of Guangxi Medical University (Nanning, China). The voucher specimen (CS-HCDL-20201015) was deposited at the Museum of Traditional Chinese Medicine Specimens of Guangxi Medical University (Nanning, China). TACS was extracted according to a previously published protocol [19] and qualitatively analyzed by UPLC-Q-TOF/MS (Supplementary Method 1).

### Animal models and drug administration

Specific pathogen-free (SPF)-grade male Sprague–Dawley rats were purchased from Changsha Tianqin Biotechnology Co., Ltd. (Changsha, China) (No. SYXK(GUI)2014-0002). All animal experiments were approved by the Animal Welfare Ethics Committee of the Guangxi Medical University (Approval No. 202111123).

**Fig.1:**
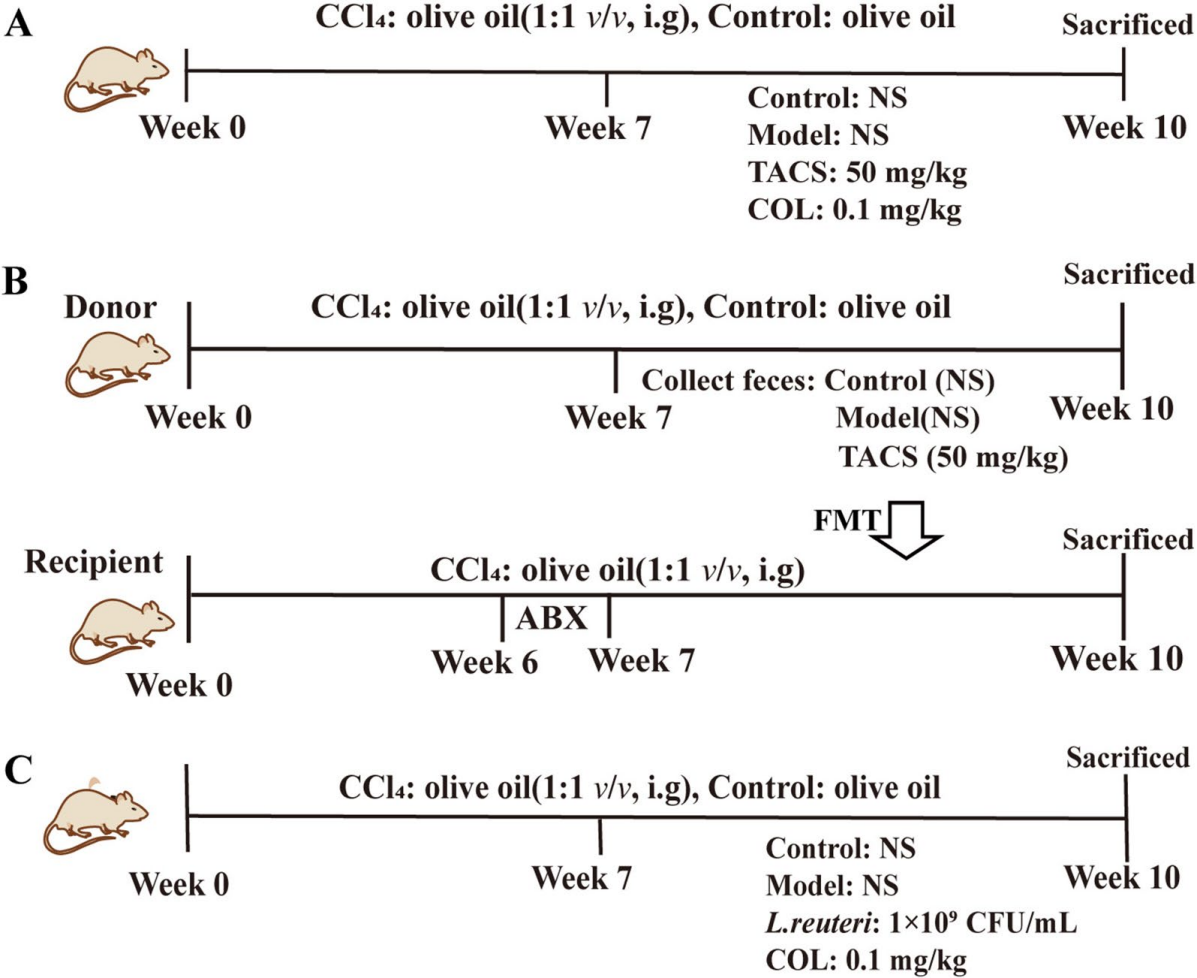
Experiment design. **A** Treatment protocols for rats. **B** FMT protocol details. **C** The *Lactobacillus reuteri* treatment protocol in rats.

All animals were housed in an SPF environment with a 12 h light/dark cycle, relative humidity of 45 ± 5%, and ambient temperature of 20-22 °C. The rats were randomly divided into the following two groups: (1) control group (*n* = 8) received olive oil (0.1 mL/100 g, i.g.); (2) CCl_4_ group (*n* = 24) received olive oil and CCl_4_ mixed (1:1 (*v*/*v*), 0.1 mL/100 g, i.g.), twice a week for 10 weeks. After 6 weeks, the CCl_4_-treated rats were assigned into the following three groups: model group (*n* = 8), TACS group (*n* = 8), and colchicine (COL) group. The TACS group rats received TACS (50 mg/kg, 0.5 mL/100 g, i.g.) and the COL group rats received colchicine (0.1 mg/kg, 0.5 mL/100 g, i.g.). The model group and control group rats received normal saline (0.5 mL/100 g, i.g.), once daily for 4 weeks (Fig. 1A).

### Antibiotic cocktail treatment

The antibiotic cocktail treatment protocol is described in Supplementary Method 2.

### Fecal microbiota transplantation

Before FMT, all the recipient rats were gavaged with antibiotic cocktail containing streptomycin (200 mg/kg), ampicillin (200 mg/kg), gentamicin (200 mg/kg), and vancomycin (100 mg/kg) for 1 week to clear the gut microbiota [20]. FMT was performed according to a previously published protocol [21]. Briefly, fecal samples were collected from the donor group rats, mainly the control, model and TACS-treated groups. 200 mg of fecal samples were suspended in sterile saline (1 mL) and centrifuged at 800 g for 3 min at 4℃. The microbial supernatants were collected and oral gavage to recipient rats (0.5 mL/100 g), once a day for four weeks (Fig. 1B).

### Oral feeding of cultured *Lactobacillus reuteri*

*Lactobacillus reuteri* (*L. reuteri*)(ATCC 23272) was obtained from the China Industrial microbial species Preservation and Management Center and grown under anaerobic conditions in Man, Rogosa and Sharpe (MRS) broth. When the culture achieved exponential growth, it was collected and stored in 50% glycerol at −80° C for further use. The *L. reuteri* culturing and growth estimation protocol is described in Supplementary Method 3. Prior to administration, a frozen sample of *L. reuteri* was thawed, resuspended in MRS, and grown. The cultures were collected in the logarithmic growth phase and washed with sterile normal saline twice. Then, the cultures were adjusted to a concentration of 1 × 10^9^ cfu/mL for gavage. LF-rats were gavaged with *L. reuteri* at a dose of 1 × 10^9^ cfu/mL (1 mL), once a day for four weeks (Fig. 1C).

### Serum biochemistry

Alanine aminotransferase (ALT), aspartate aminotransferase (AST), and alkaline phosphatase (ALP) concentrations were quantified using the HITACHI automatic biochemical analyzer 7100 (Hitachi Ltd.,Tokyo, Japan).

### Estimation of LPS and pro-inflammatory cytokine levels

The levels of LPS, TNF-α, IL-6 and IL-1β in the liver samples from distinct groups of rats were quantified using the ELISA kits (Shanghai Fanke Industrial Co. LTD) according to manufacturer’s instructions.

### Histopathological analysis

Colon and liver tissues from rats were fixed in 4% paraformaldehyde, paraffin-embedded and sectioned into 4 μm-thick slices. Then, the sections were stained with the Masson’s trichrome and H&E stains to visualize the tissue structural changes.

### Immunohistochemistry (IHC)

IHC assay was performed according to a previously described protocol [19]. The liver tissue sections were stained with the anti-α-SMA and anti-COL1A1 antibodies and examined using the MT52-N microscope (MSHOT, Guangzhou, China).

### Metabolomics analysis of bile acids in cecal contents and liver samples

The ultra performance liquid chromatography coupled with triple quadrupole mass spectrometry (UPLC-MS/MS, Waters Corp., Milford, MA, USA) was performed as described previously to determine the BA profiles in the rat cecal contents and liver samples [22].

### 16S rRNA gene sequencing

The E.Z.N.A. Stool DNA Kit (D4015, Omega, Inc., USA) was used to extract the total bacterial genomic DNA in the cecum and fecal samples of rats from all the study groups. The V3-V4 region of the 16S rRNA gene was amplified using the forward (5'-CCTACGGGNGGCWGCAG-3') and reverse (5'-GACTACHVGGGTATCTAATCC-3') primers. The PCR products were sequenced using the Illumina HiSeq 2500 platform.

### Metagenomic analysis

The cecum samples were sequenced using the NovaSeq/Hiseq X ten platform (Illumina Inc., San Diego, CA, USA). The data were analyzed on the online platform of Majorbio Cloud Platform (www.majorbio.com).

### Statistical analysis

Statistical analysis was performed using the SPSS 20.0 (IBM SPSS, USA) and GraphPad Prism (San Diego, USA) software. The data were represented as mean ± SEM. Differential analysis was performed using the one-way ANOVA, Mann–Whitney U test, and Wilcoxon rank-sum test. *P* < 0.05 was considered as statistically significant.

## Results

### Qualitative analysis of chemical compounds in TACS

**Fig. 2:**
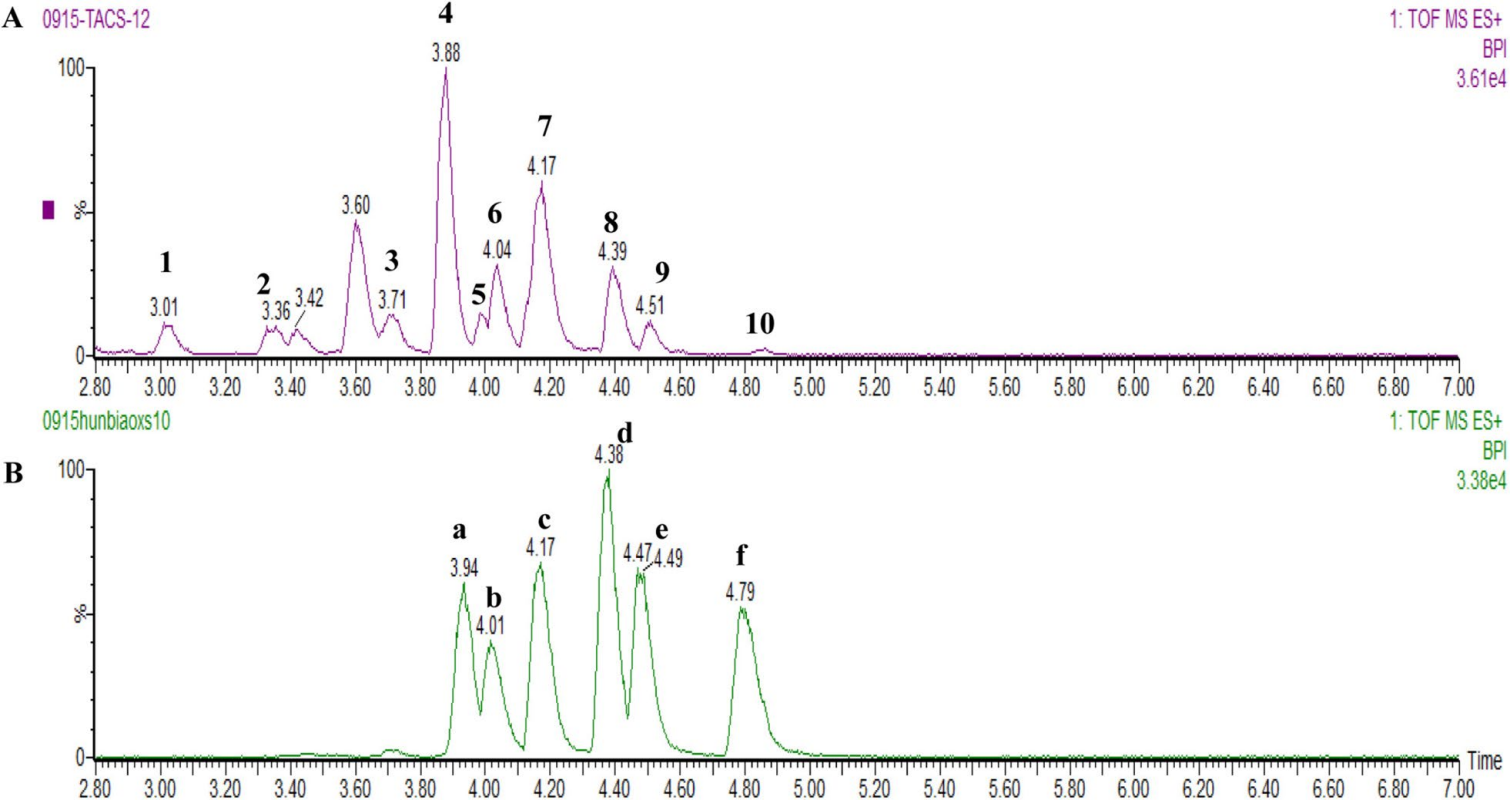
Base peak ion chromatograms of **A** TACS and **B** mixed standard samples based on UPLC-Q-TOF/MS(ESI +). The following chemical components were identified in TACS: (1) Cheilanthifoline; (2) Berberrubine; (3) Epiberberine; (4) Tetrahydropalmatine; (5) Jatrorrhizine; (6) Coptisine; (7) Dehydrocavidine; (8) Palmatine; (9) Berberine; (10) Chelerythrine. Standard: a: Jatrorrhizine; b: Coptisine; c: Dehydrocavidine; d: Palmatine; e: Berberine; f: Chelerythrine

The base peak ion chromatograms for TACS and mixed standard solutions are shown in Fig. 2. We identified 10 compounds in TACS, including berberrubine, cheilanthifoline, tetrahydropalmatine, epiberberine, jatrorrhizine, dehydrocavidine, coptisine, berberine, palmatine, and chelerythrine (Fig. S1 and Table S1). The fragmentation patterns of the compounds in TACS are shown in the Supplementary Materials.

### TACS significantly alleviates CCl_4_-induced LF in rats

**Fig. 3:**
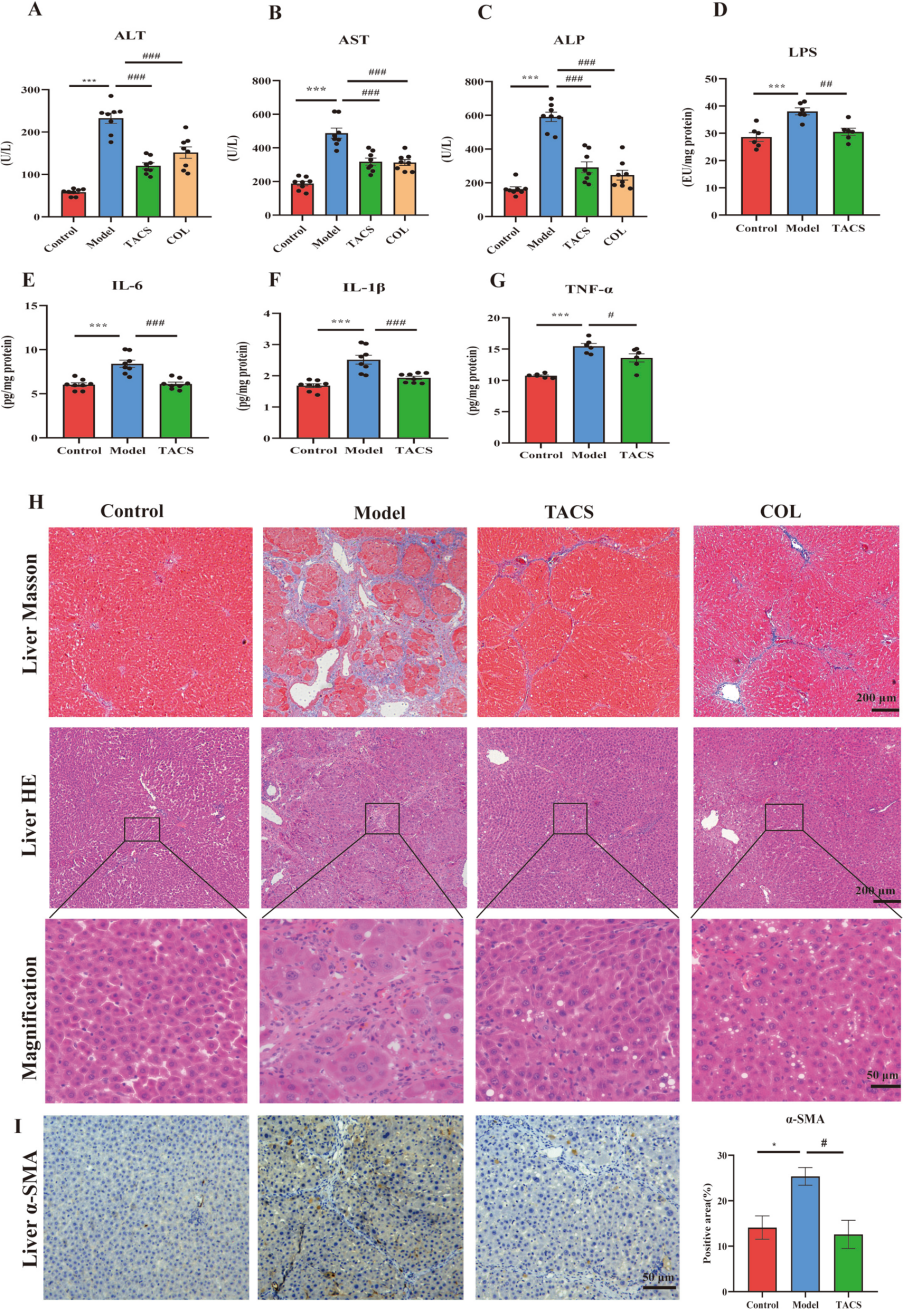
Effects of TACS on CCl_4_-induced liver fibrosis model rats. (**A**–**C** Levels of AST, ALT and ALP in serum(*n* = 8). **D** The levels of LPS in the livers (*n* = 6). **E**–**G** The levels of IL-6, IL-1β, and TNF-α in the liver (*n* = 6–8). **H** Representative images of liver tissues stained with Masson and H&E stains. **I** IHC analysis shows α-SMA levels in the liver tissues (*n* = 3). Data are expressed as mean ± SEM; *P < 0.05, ***P < 0.001 vs. control group; ^#^*P* < 0.05, ^##^*P* < 0.01, and ^###^*P* < 0.001 *vs* model group

We established LF model rats to investigate the therapeutic effects of TACS. The serum levels of ALT, AST, and ALP were significantly lower in the TACS group rats compared to the LF model group rats (Fig. 3A–C). TACS group rats also showed lower levels of LPS, IL-6, TNF-α, and IL-1β in the liver compared to the model group rats (Fig. 3D–G). TACS administration significantly reduced fibrosis in portal tracts, necrosis of liver cells and inflammatory infiltration in liver tissues compared to the model group rats (Fig. 3H). IHC results demonstrated that liver α-SMA level was significantly higher in response to CCl_4_ stimulation but were reduced by TACS treatment (Fig. 3I). These data indicate that TACS has anti-LF effect.

### TACS regulates bile acid metabolism in the LF model rats

**Fig. 4:**
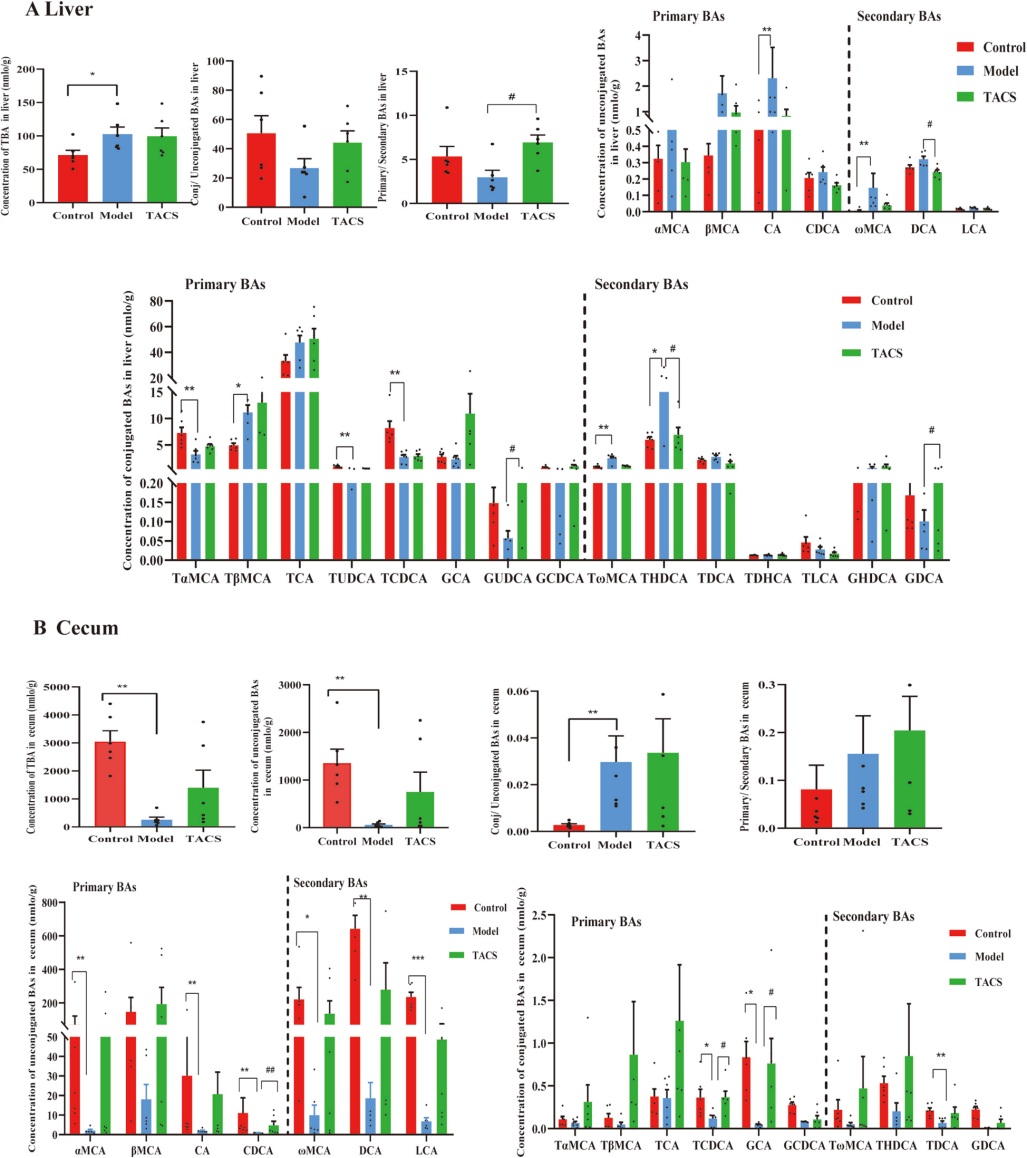
TACS treatment alters the levels of bile acids in the liver and cecum of the LF model rats. **A** The profiles of different BA classes in the liver samples (*n* = 6). **B** The profiles of different BA classes in the cecal contents samples (*n* = 6). Data are expressed as mean ± SEM; *P < 0.05, ^**^P < 0.01, and ^***^P < 0.001 *vs*. control group; ^#^*P* < 0.05 *vs* model group

To further study the effects of TACS on the liver and cecum in the LF model rats. BA targeted metabolomics method was performed for examine the BAs in liver and cecal contents of rats. 41 and 40 bile acids were detected in liver and cecum, respectively (Table S2, Table S3). The principal component analysis (PCA) showed that the metabolic profile of BAs after TACS treatment tended to be closer to the control group and was distinctly separated from the model group (Fig. S2). In the liver of the model group rats, the levels of total BAs, ω-muricholic acid(ωMCA), tauro β-muricholic acid(TβMCA), and CA were significantly higher and the levels of tauro α-muricholic acid (TαMCA) and tauroursodeoxycholic acid (TUDCA) were significantly lower compared to the control group rats. However, the levels of DCA, glycoursodeoxycholic acid (GUDCA), taurohyodeoxycholic acid (THDCA), and glycodeoxycholic acid (GDCA) in the liver of the TACS treatment group were significantly different compared to the model group rats (Fig. 4A). In the cecal samples of model group rats, the concentration of total BAs was significantly reduced, and the ratio of conjugated BAs/unconjugated BAs was significantly increased compared to the control group rats. Moreover, compared with the control group rats, the levels of ωMCA, α-muricholic acid(αMCA), CA, DCA, TCDCA, glycocholic acid (GCA), CDCA, and lithocholic acid(LCA) were significantly lower in the model group rats. However, the levels of GCA and CDCA were significantly increased in the TACS treatment group compared to the model group (Fig. 4B). This demonstrated that TACS treatment significantly altered the bile acid profile in the LF rats.

### TACS alleviates liver fibrosis -induced gut dysbiosis in rats

**Fig. 5:**
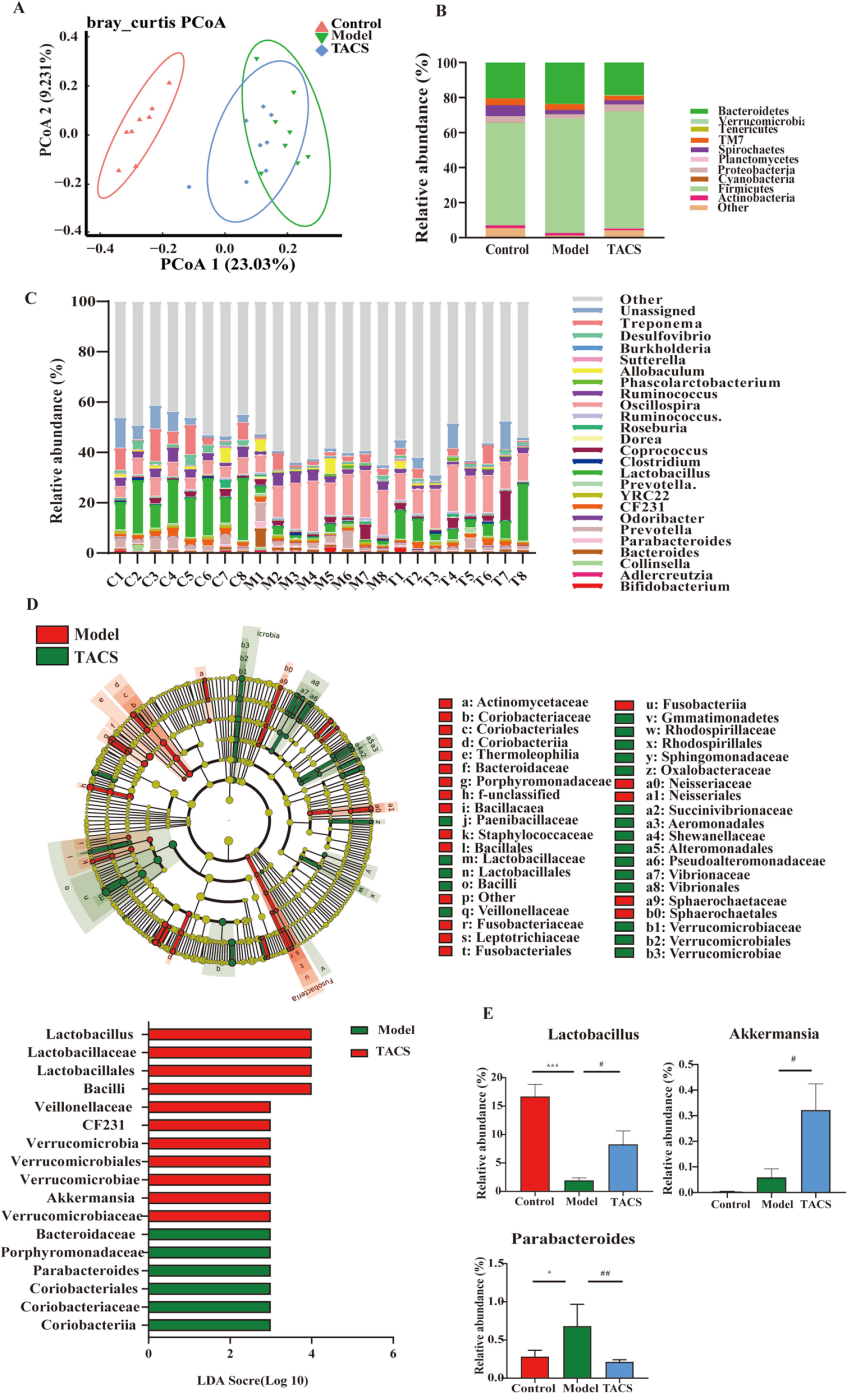
TACS alleviates CCl_4_-induced gut dysbiosis. The composition of cecal microbiota in each group of rats was analyzed by 16S rRNA gene sequencing. **A** Principal coordinate analysis (PCoA) based on weighted Bray–Curtis index shows changes in the gut microbial diversity between the control, model, and TACS-treated rat groups. **B** Relative abundances of bacterial phyla in different groups rats. **C** Relative abundances of gut microbiota genera in the control, model, and TACS-treated rat groups. **D** Evolutionary branch diagram shows the LEfSe analysis of the gut microbiota in the control, model, and TACS-treated rat groups. **E** Relative abundances of *Lactobacillus*, *Akkermansia* and *Paraprevotellaceae* in the control, model, and TACS-treated rat groups (*n* = 6)*.* Data are expressed as mean ± SEM; ^*^P < 0.05, ^***^P < 0.001 *vs*. control group, ^#^*P* < 0.05, ^##^*P* < 0.01*vs* model group

In previous studies, animal models of LF have also demonstrated dysbiosis of the gut microbiota. Therefore, to investigate the effects of TACS on the overall structure of gut microbiota in the LF rats, 16S rRNA gene sequencing was applied to detected cecal content from the control, model, and TACS groups of rats. First of all, the specaccum accumulation curves suggested the sufficient species abundance for each sample (Fig. S3A). As shown in Fig. S3B-C, there were significant changes in the Simpson and Shannon indices between the 3 groups, thereby indicating changes in the α-diversity of the gut ecosystem. Principal coordinate analysis (PCoA) based on weighted Bray–Curtis distances showed significant differences in the gut microbial profiles between the control and model groups, but these changes were partially alleviated by TACS (Fig. 5A). Compared with the model group rats, phylum-level analyses demonstrated that the abundance of *Bacteroidetes* phylum was increased after TACS treatment, whereas no obvious differences (Fig. 5B). At the genus level, the relative abundance of *Lactobacillus* increased significantly in the TACS group rats compared to the model group rats (Fig. 5C). The LEfSe analyses demonstrated that the relative abundances of *Lactobacillaceae*, *Lactobacillus*, *Veillonellaceae*, *Paraprevotellaceae,* and *CF231* were reduced by CCl_4_ stimulation (Fig. S3D), but TACS treatment increased the abundances of *Lactobacillaceae, Lactobacillus*, *Veillonellaceae*, *CF231,* and *Akkermansia* genus (Fig. 5D-E). The colon histopathology of the LF rats exhibited loss of crypt and epithelial cells, but these changes were significantly alleviated by TACS (Fig. S3E). These data demonstrated that TACS treatment significantly mitigated the gut microbiota disorder in the LF rats.

### TACS mediates enrichment of *L. reuteri* and significantly alters bile acid metabolism in the gut microbiota of the LF model rats

**Fig. 6:**
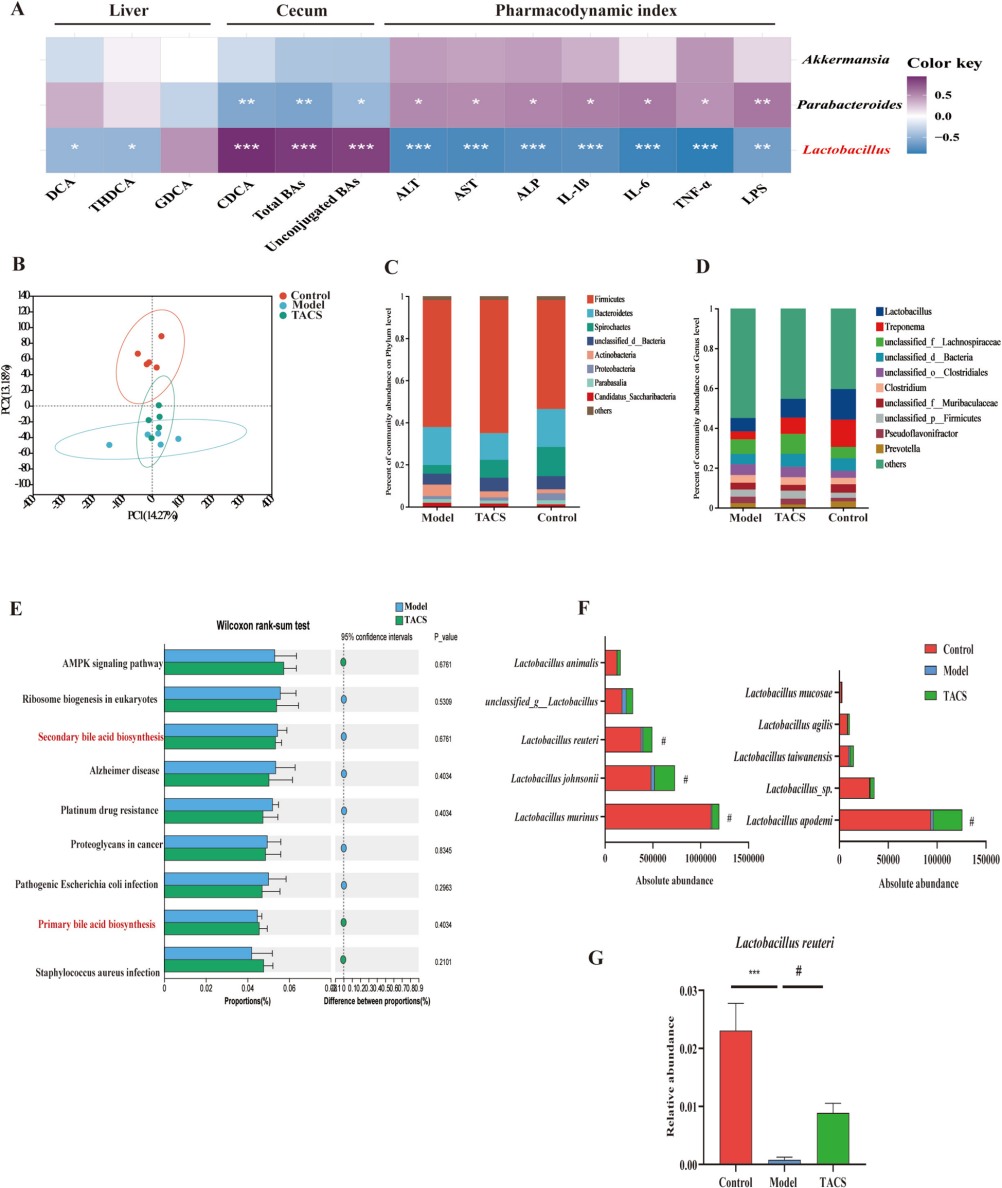
TACS mediates enrichment of *L. reuteri*. **A** Spearman’s correlation analysis shows the relationships between gut microbiota, BAs, and liver fibrosis-related parameters in different rat groups. Purple represents positive correlation, and blue indicates negative correlation. ^*^P < 0.05, ^**^P < 0.01, ^***^P < 0.001. **B** The composition of cecal microbiota in each group of rats was analyzed by metagenomics sequencing analysis. PCA shows changes in the gut microbial diversity between the control, model, and TACS-treated rat groups. **C** The relative abundance of cecal microbiota phyla in rats of different groups. **D** Relative abundances of cecal microbiota genera in rats of different groups. **E** The functional profiles were identified by metagenomic analysis utilizing both KEGG databases. **F** Absolute abundance of various *Lactobacillus* species in the cecal contents samples of rats based on metagenomics sequencing analysis. (^#^*P* < 0.05 *vs* model group, *n* = 5). **G** Relative abundance of *L. reuteri* rats. Data expressed as mean ± SEM *(n* = 5); ^*^P < 0.05, ^**^P < 0.01 vs. control group; ^#^*P* < 0.05 *vs* model group

Based on the above results, Spearman’s correlation analysis was performed to assess the relationship of the liver fibrosis-related parameters with key gut microbiota and key BAs. Our data showed that *Lactobacillus* was the most abundant genus. The abundance of *Lactobacillus* negatively correlated with most of the LF-related parameters such as serum AST, ALT, and ALP levels, pro-inflammatory cytokine levels in the liver, and levels of DCA and THDCA in the liver, but positively correlated with the levels of CDCA, total BAs, and unconjugated BAs in the cecum (Fig. 6A). This indicated that bacteria in the *Lactobacillus* genus correlated with the levels of BAs and the anti-LF activity of TACS. Then, we performed metagenomics to further investigate the changes in the abundances of different *Lactobacillus* species. PCA demonstrated that the gut microbiota spectrum of the control and model groups were clearly separated and the TACS group was closer to the control group (Fig. 6B). At the phyla level, *Firmicutes* and *Bacteroidetes* accounted for more than 90% of gut microbiota (Fig. 6C). Moreover, TACS altered the level of *Lactobacillus* (Fig. 6D). This was consistent with the 16S rRNA gene sequencing results. Next, we further studied the changes in the functional characteristics of intestinal microbiota after TACS treatment. Kyoto Encyclopedia of Genes and Genomes (KEGG) database was used to analyze the metagenomic sequencing data to predict the function of the gut microbiota in the LF model rats. The functional changes in the gut microbiota of rats were mainly associated with the AMPK signaling pathway, primary bile acid metabolism, and secondary bile acid metabolism (Fig. 6E). Among the top 10 abundant species, *L. reuteri* was associated with LF and showed significant enrichment after TACS treatment compared to the model group (Fig. 6F–G). These results suggested that gut microbiota dysbiosis in the LF model rats was closely related with bile acid metabolism.

### The anti-LF effects of TACS are associated with gut microbiota

To determine whether the anti-LF effects of TACS were dependent on the gut microbiota, we treated LF model rats with a cocktail of antibiotics to reduce most of the gut microbiota. The anti-LF effects of TACS were not observed when the gut microbiota was inhibited by antibiotics. We did not observe statistically significant differences in the serum AST, ALT, and ALP levels (Fig. S4A–C), and the levels of pro-inflammatory factors in the liver between the antibiotics and antibiotics + TACS groups(Fig.S4D-F). Furthermore, TACS treatment-related benefits including reduced levels of liver collagen fiber and increased number of intestinal epithelial cells was attenuated by antibiotic intervention (Fig. S4G–I). Together, these data suggested that the anti-LF effects of TACS were associated with the gut microbiota.

### Fecal transplantation from TACS rats ameliorates liver fibrosis

**Fig. 7:**
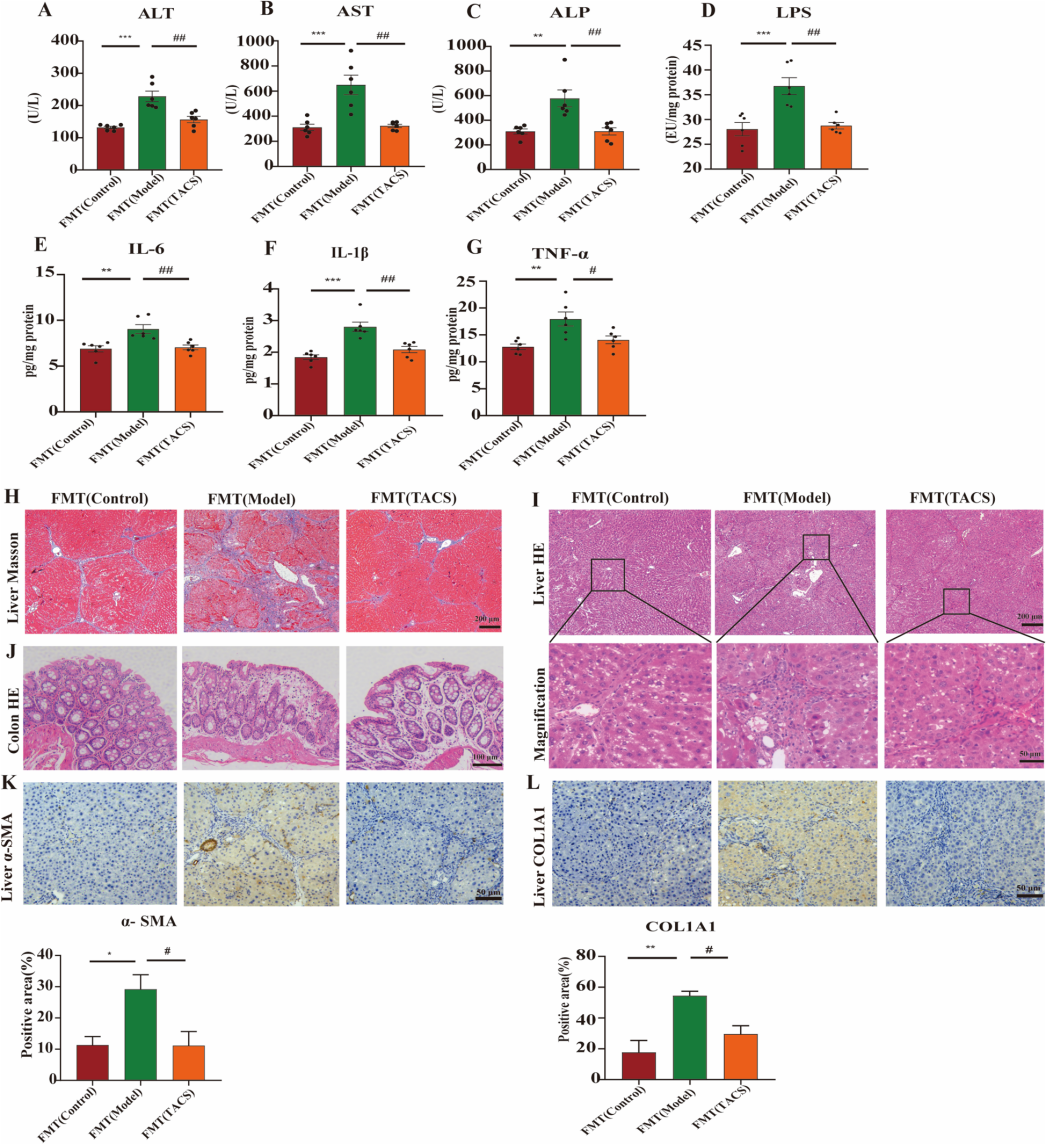
Fecal microbial transplantation from TACS rats ameliorates liver fibrosis in the model rats. **A**–**C** Serum levels of ALT, AST, and ALP in FMT(Control), FMT(model), and FMT(TACS) rat groups. (*n* = 6). **D**–**G** Levels of LPS, IL-6, IL-1β and TNF-α in the liver tissues of FMT(Control), FMT(model), and FMT(TACS) rat groups (*n* = 6). **H**, **I** Representative Masson and H&E-stained liver tissue sections from different rat groups. **J** Representative H&E-stained colon tissue sections from different rat groups. **K** IHC staining analysis shows the levels of α-SMA in the liver tissues of different rat groups (*n* = 3). **L** IHC staining analysis shows the levels of COL1A1 in the liver tissues of different rat groups (*n* = 3). Data are expressed as mean ± SEM; ^**^P < 0.01, ^***^P < 0.001 *vs*. FMT(Control) group; ^#^*P* < 0.05, ^##^*P* < 0.01 *vs* FMT(model) group

To further confirm that the gut microbiota mediate the anti-LF effects of TACS, we transplanted gut microbiota from control, model, and TACS group rats to recipient rats and analyzed the LF-related traits. Four weeks after transplantation of the fecal microbiota, the FMT (TACS) rats demonstrated significantly lower serum ALT, AST, and ALP levels (Fig. 7A–C), as well as the liver LPS, IL-1β, IL-6, and TNF-α levels compared to the FMT (Model) rats (Fig. 7D–G). Histopathological analysis of the liver showed that the liver collagen fiber area and hepatocyte necrosis was significantly lower in the FMT (TACS) rats compared to the FMT (Model) rats (Fig. 7H–I). Besides, fecal transfer from the TACS rats significantly ameliorated the pathological changes in the colon tissue (Fig. 7J). Moreover, fecal transfer from TACS rats decreased α-SMA and COL1A1 expression levels in the liver tissues of the FMT (TACS) rats compared to the FMT (Model) rats (Fig. 7K, L). To confirm that fecal microbiota transplantation modulates gut microbiota, 16S rRNA gene sequencing was used to analyze the composition of fecal gut microbiota in FMT rats. PCoA demonstrated distinct separation in the gut microbiota communities between the FMT (Control) group and the FMT (Model) group, with a tendency for FMT(TACS) to approach FTM (Control)(Fig.S5A). The results at the phyla level showed that the proportion of *Firmicutes* and *Bacteroides* in the gut of FMT rats was more than 90%(Fig.S5B). At the genus level, the relative abundance of *Lactobacillus* was increased in the FMT(TACS) group rats compared to the FMT(model) group rats(Fig.S5C-D). Collectively, these results suggested that the anti-LF effects of TACS were mediated through the gut microbiota.

**Fig. 8:**
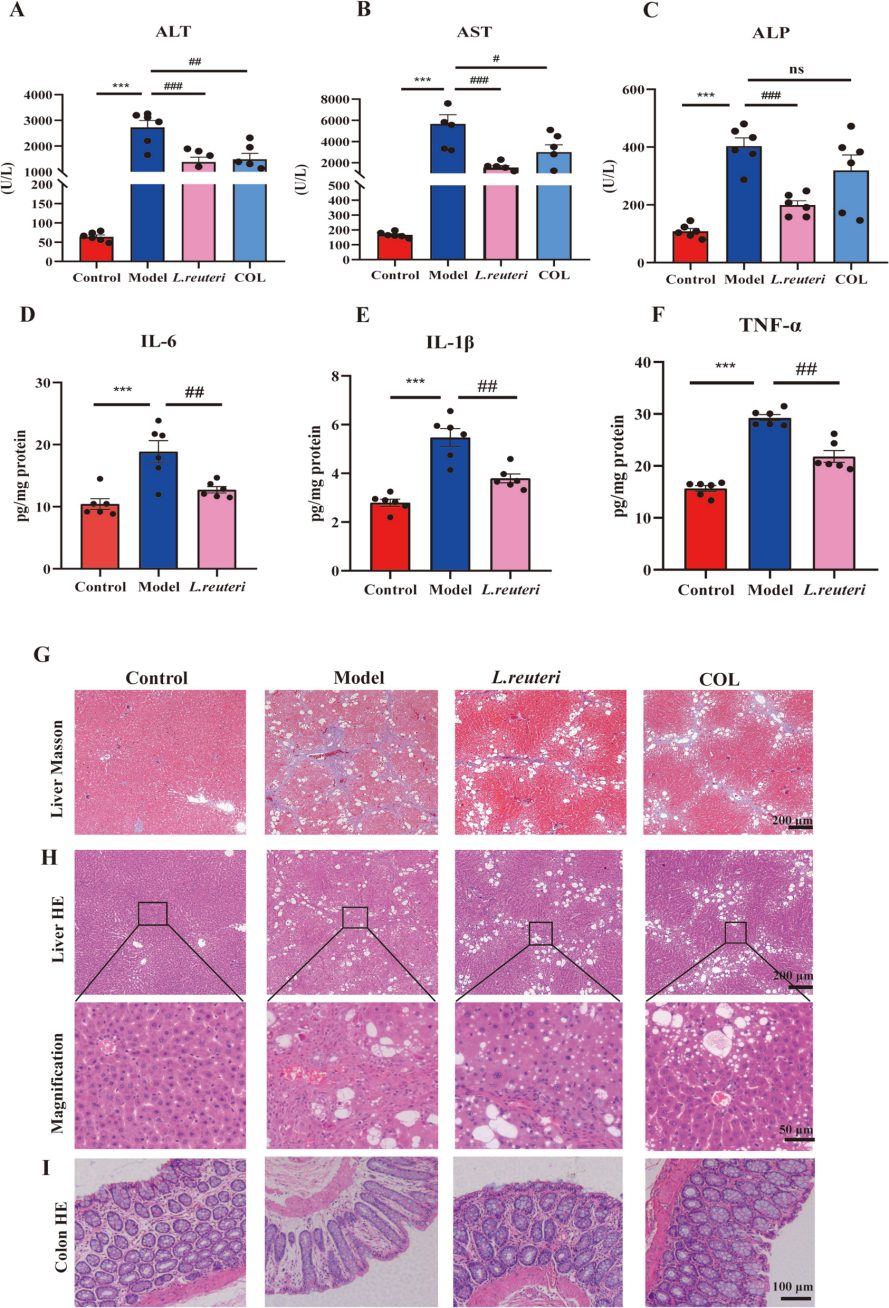
The anti-liver fibrosis effects of *Lactobacillus reuteri.* **A**–**C** Serum levels of AST, ALT, and ALP in the control, model, and *L. reuteri* rat groups. (*n* = 6). **D**–**F** Levels of IL-6, IL-1β, and TNF-α in the liver tissues of different rat groups (*n* = 6). **G**–**H** Representative images show Masson trichome and H&E-stained liver tissue sections from different rat groups. **I** Representative images show H&E-stained colon tissue sections from different rat groups. Data are expressed as mean ± SEM; ^*^P < 0.05, ^***^P < 0.001 *vs* control group; ^*#*^*P* < 0.05, ^##^*P* < 0.01, ^###^*P* < 0.001 *vs* model group

### *L. reuteri* improves CCl_4_-induced liver fibrosis in rats

As shown in Fig. 6G, we identified *L. reuteri* as a critical bacterial species responding to TACS treatment. Therefore, we assumed that *L. reuteri* mediated the anti-LF effects. To test this assumption, LF rats were gavaged daily with *L. reuteri* for 4 weeks and subsequently analyzed the LF-related traits. Compared to the model rats, *L. reuteri* significantly reduced various LF-related indicators, including serum levels of AST, ALT, and ALP(Fig.8A-C), pro-inflammatory factors in the liver(Fig.8D-F), collagen fiber deposition in the liver, hepatic steatosis and inflammatory infiltration, and histological damage in the colon tissues (Fig. 8G–I). Together, these results demonstrated that *L. reuteri* protected the intestinal barrier and significantly alleviated liver fibrosis induced by CCl_4_.

## Discussion

Liver fibrosis is a significant health problem worldwide [23]. Several studies have shown that bile acids and gut microbiota play an important role in the prevention and treatment of liver fibrosis [24]. Given that the liver and intestine are identical at embryonic origin and maintain natural physiological functional links, the contribution of the liver-axis cannot be ignored [25]. Therefore, the regulation of bile acids and gut microbiota is critical for understanding the potential mechanisms underlying liver fibrosis and discovering novel and effective interventions. The research aimed to investigate the anti-liver fibrosis effect of TACS from the perspective of bile acid metabolism and gut microbiota. LF model rats were generated by administration of CCl_4_ as described in previous studies [19, 26]. The intragastric administration modeling approach demonstrates distinct advantages in liver fibrosis research, including operational simplicity, enhanced drug bioavailability, superior model stability, and reduced animal distress. This method is suitable for long-term experimental protocols (e.g., chronic liver fibrosis or cirrhosis models exceeding 8 weeks) requiring maintained high survival rates in animal model systems [27, 28].TACS are the main active component of *Corydalis saxicola* Bunting, a traditional Chinese herbal formulation. TACS exhibit anti-inflammatory, antiviral, and liver protective effects [29]. In this study, we characterized the chemical constituents of TACS using UPLC-Q-TOF/MS and identified 10 chemical components. This included berberrubine [30], tetrahydropalmatine [31], jatrorrhizine [32], coptisine [33], berberine [34], and chelerythrine [35], which have been shown previously to be effective in the treatment of liver diseases. Furthermore, dehydrocavidine and palmatine exhibit anti-LF effects [26, 36]. Previous studies have shown that TACS alleviate gut microbiota dysbiosis in rats with antibiotic diarrhea [17]. TACS also significantly alleviates liver injury and fibrosis in rats [18, 19]. Our results demonstrate that administration of TACS for four weeks improve liver fibrosis in the LF model rats. TACS treatment significantly reduces the serum levels of ALT, AST, and ALP. Microbiological and metabonomic analyses demonstrated that TACS alleviates abnormal bile acid metabolism and gut microbiota imbalance in the LF model rats. Correlation analysis showed a close relationship between bile acid metabolism and *Lactobacillus* in the gut microbiota disorder of the LF model rats. Metagenomic data further confirmed the link between dysregulation of bile acid metabolism and gut microbiota in the LF model rats and demonstrated that TACS significantly reverses the relative abundance of *Lactobacillus reuteri.* Therefore, TACS alleviates liver fibrosis by regulating bile acid metabolism and gut microbiota. The results of the antibiotic cocktail treatment and fecal microbiota transplantation (FMT) experiments demonstrated that the anti-LF effects of TACS were related to gut microbiota. *L. reuteri* was a key player in improving LF. These findings provide new insights for the development of anti-LF drugs and probiotics.

LF is closely related with significant changes in the BA levels [2]. Previous studies have shown that the accumulation of BAs in the liver contribute to the development of liver fibrosis [37]. In our study, the LF model rats showed elevated levels of total BAs and CA, but TACS treatment significantly reduced these levels. CA is a hydrophobic bile acid that induces oxidative stress and inflammation in the liver cells, thereby aggravating LF [38]. LF model rats also showed significantly high levels of liver inflammatory factors (TNF-α, IL-6, and IL-1β), but TACS treatment reversed these effects. Hepatocytes activate HSCs by internalizing IL-1β and releasing the Caspase-11 pyroptosome [3, 39]. Furthermore, CDCA and DCA also induce pyroptosis by activating Caspase-11 through a non-canonical pathway involving mitochondrial permeability transition (MPT) [40]. Our results demonstrated that the levels of DCA, α-SMA, COL1A1, and proinflammatory cytokines were decreased in the TACS treatment group. This suggested that TACS might inhibit the activation of HSCs by reducing liver DCA levels. This alleviated liver fibrosis by reducing the levels of α-SMA, COL1A1, and inflammatory factors. CDCA is a potent endogenous bile acid and FXR agonist. CDCA stimulates FXR-FGF19 signaling in the hepatocytes and activates FGFR4 signaling in the liver. This inhibits bile acid synthesis and reduces the accumulation of bile acids in the liver [7]. In our study, TACS significantly increased the levels of CDCA in the intestinal tract of the LF model rats. This suggested that TACS might reduce liver fibrosis by increasing CDCA levels, activating the FXR-FGF15 signaling pathway, and inhibiting liver bile acid synthesis, thereby reducing the accumulation of BAs in the liver. However, there are still some limitations in this study. In current study, bile acids profile was analyzed and emphasized on their role in model and TACS therapy, but no direct exploration on whether the altered bile acids contributed to LF attenuation by TACS. In future studies, bile acid administration therapy and the combination of bile acid receptor-specific inhibitors or agonists were used to deeply explore the molecular mechanisms involved in the formation or attenuation of LF caused by the altered bile acids after TACS treatment.

Gut microbiota dysbiosis is an important driving factor of liver fibrosis through the liver-gut axis [24, 41]. Gut microbiota dysfunction aggravates liver injury by inducing intestinal inflammation and barrier damage [42]. In our study, LF model rats showed damaged intestinal barrier and increased permeability, but TACS significantly alleviated intestinal tissue damage. In the multidrug-resistant protein (Mdr) knockout mice, gut microbiota dysbiosis accelerated the progression of liver disease [43]. Supplementation with *Lactobacillus acidophilus* promotes recovery of liver function [25]. *Lactobacillus rhamnosus* GG prevents liver fibrosis by activating the expression of nuclear factor erythrocyte derived 2-like 2 (Nrf2) in the liver [7]. In this study, antibiotic cocktail treatment and FMT confirmed that the anti-LF effects of TACS were mediated by the gut microbiota. LF model rats transplanted with feces from the TACS group rats showed decreased LF. When antibiotics were used to inhibit gut microbiota, the anti-LF effects of TACS were significantly reduced. Our results demonstrated that attenuation of LF by TACS was associated with the abundances of *Lactobacillus, Akkermansia,* and *Paraprevotellaceae*. TACS treatment significantly enriched the relative abundance of *Akkermansia*, which degrades mucoprotein. The relative abundance of *Akkermansia* is reduced in the fecal samples of liver cirrhosis patients [44]. Furthermore, the relative abundance of *Paraprevotellaceae* was significantly reduced by TACS treatment. *Paraprevotellaceae* is significantly increased in patients with liver disease [45]. TACS also increased the relative abundance of *Lactobacillus*, which is a probiotic that improves liver fibrosis and inflammation [46]. *Lactobacillus* species are bile salt hydrolase (BSH)-enriched bacterium and are involved in BA metabolism [7].

**Fig. 9:**
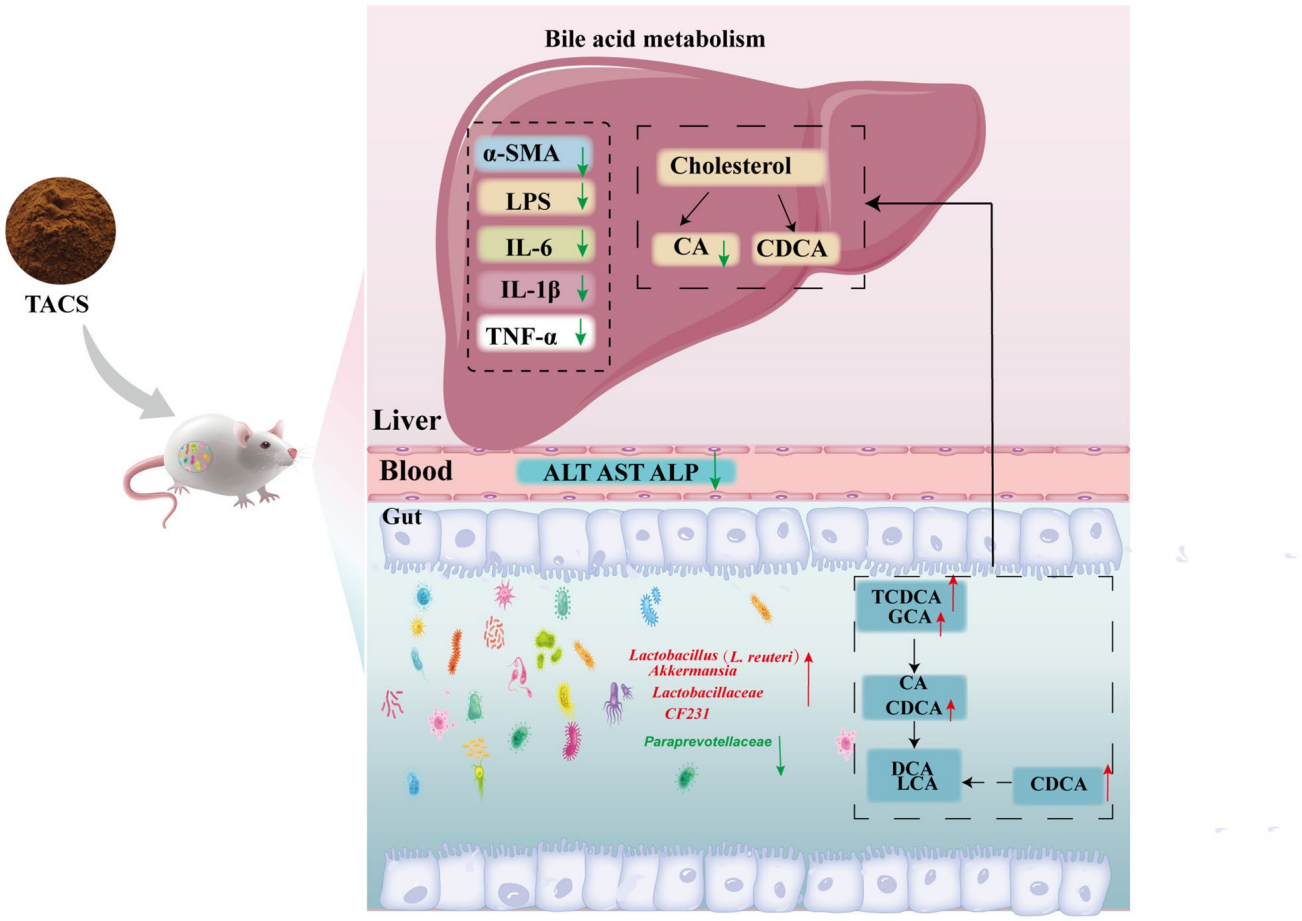
TACS attenuates CCl_4_-induced liver fibrosis in rats by modulating BA metabolism and gut microbiome. Compared to the model group, the red arrow indicates increase in the TACS group and the green arrow indicates decrease in the TACS group.

The gut microbiota interacts with the host by producing several metabolites, including BAs. BSH-producing bacteria adjust the BA pool by deconjugating BA, thereby affecting bile acid metabolism [47]. Spearman’s correlation analysis demonstrated significant association between *Lactobacillus* and BAs. *Lactobacillus* attenuates excessive BA-induced LF by increasing the population of BSH-producing bacteria and promoting the excretion of BAs in the feces [7]. TACS treatment increased BA levels in the cecum. This suggested that TACS promotes the enrichment of BSH-producing bacteria (*Lactobacillus*), thereby resulting in increased levels of CA, DCA, and CDCA. Metagenomic results also showed that the gut microbiota modulates both primary and secondary bile acid metabolism. Therefore, we hypothesized that TACS alleviates hepatic fibrosis by regulating bile acid metabolism (Fig. 9).

Drugging the microbiome is a rapidly developing field with significant potential for therapeutic applications [48]. In this study, FMT and antibiotic cocktail treatment confirmed that TACS alleviates LF by regulating gut microbiota. We then focused on the significantly enriched bacteria in the TACS group compared to the LF model group. *Lactobacillus* has shown significant correlation with bile acids and pharmacodynamic indices. Furthermore, metagenomic analysis demonstrated that *L. reuteri* was highly enriched after TACS treatment. *L. reuteri* alleviates liver injury by regulating BA metabolism [49]. However, there is no direct evidence for the role of *L. reuteri* in LF. Therefore, we used a strain of *L. reuteri* (ATCC 23272) in the animal experiments. Our results demonstrated that oral administration of *L. reuteri* significantly reduced the serum levels of AST, ALT, and ALP, and the levels of inflammatory factors in the liver of the LF model rats. These data further confirmed that TACS alleviates LF by modulating the gut microbiota, including enrichment of *L. reuteri*, a type of intestinal probiotic bacteria. However, there are some limitations in this study. Although this study demonstrates the effects of TACS on bile acid metabolism and gut microbiota, further investigations are necessary to determine the in-depth molecular mechanisms involved in the process. Furthermore, investigations at the cellular and molecular levels are necessary to determine the precise mechanisms by which TACS modulates the abundance of *L. reuteri* and bile acid metabolism.

## Conclusions

This study demonstrates that TACS alleviates LF by reducing the levels of pro-inflammatory cytokines in the liver, improving serum liver biochemical indicators, and modulating bile acid metabolism and gut microbiota. Antibiotic cocktail treatment and FMT results suggested that the anti-LF effects of TACS were closely associated with the gut microbiota. Furthermore, *L. reuteri* reduces the levels of pro-inflammatory cytokines and serum biochemical indicators and ameliorates impaired liver tissue lesions. In summary, TACS exerts anti-LF effects by regulating bile acid metabolism and gut microbiota. These findings provide an important reference for studying the mechanism of TACS anti-liver fibrosis, and have important guiding significance for the future development and application of TACS-based drug formulations and prebiotics against liver fibrosis. However, there are still some limitations in this study. Although this study confirmed the effects of TACS on bile acid metabolism and intestinal microbiota in LF rats, it did no direct exploration on whether the altered bile acids contributed to LF attenuation by TACS. In future studies, we will combine bile acid receptor agonists or inhibitors to investigate the exact mechanism by which TACS regulate bile acid metabolism and gut microbiota to alleviate LF at the animal, cellular and molecular biology experiments.

## Supplementary Information

Additional file 1

Additional file 2

## Data Availability

Data will be made available upon reasonable request.

## Abbreviations

ALP: Alkaline phosphatase
BA: Bile acid
CA: Cholic acid
ECM: Extracellular matrix
FMT: Fecal microbiota transplantation
FXR: Farlactone X receptor
HSC: Hepatic stellate cells
KEGG: Kyoto Encyclopedia of Genes and Genomes
LF: Liver fibrosis
MRS: Man, Rogosa and Sharpe
PCA: Principal component analysis
PCoA: Principal coordinate analysis
TACS: Total alkaloids of *Corydalis* *saxicola Bunting*
TCM: Traditional Chinese medicine
