# Supplementary material for: Integration bile acid metabolomics and gut microbiome to study the anti-liver fibrosis effects of total alkaloids of Corydalis saxicola Bunting
Source: Chin Med. 2025 Jul 4;20:106. doi: 10.1186/s13020-025-01158-2 (PMC12226865; doi:10.1186/s13020-025-01158-2)
Supplement: Supplementary file 2 — Additional file 2 [file 13020_2025_1158_MOESM2_ESM.docx]

**Supplementary Materials and Methods**

**Integration bile acid metabolomics and gut microbiome to study the anti-liver fibrosis effects of total alkaloids of *Corydalis saxicola* Bunting**

**Supplementary methods**

**Method 1.**

**1. Qualitative analysis of the chemical components in TACS**

**1. Materials and methods**

**1.1 reagents**

Coptisine (Lot number: MUST-14051612), berberine (Lot number: MUST-16111115), palmatine (Lot number: MUST-17022604), chelerythrine (Lot number: MUST-14062313), epiberberine (Lot number: MUST-17072011) and jatrorhizine(Lot number: MSUT-17041801) were purchased from Chengdu Must Bio-Technology Co., Ltd (Chengdu, China). Dehydrocavidine (Lot number: 11667-200401) was purchased from National Institute for the Control of Pharmaceutical and Biological Products (Beijing, China).

**1.2 Sample preparation**

The TACS was dissolved with methanol to obtain a 0.5 mg/ml solution of TACS. The mixed standard solution contained a mixture of coptisine, jatrorhizine, dehydrocavidine, palmatine, berberine and chelerythrine at a concentration of 0.1 mg/ml.

**1.3 UPLC-Q-TOF/MS analysis**

Chromatographic separation was performed with an Acquity UPLC HSS T3 column (100 mm × 2.1 mm, 1.8 μm) using the Waters ACQUITY UPLC system (Waters Corp. Milford, USA) maintained at 40 °C. Acetonitrile (A) and water with 0.1% formic acid (B) were used as the mobile phases. The flow rate was set at 0.5 ml/min. The gradient elution conditions were as follows: 0-0.3 min, washing with 90% A; 0.3-1.0 min, 90% A to 80% A; 1.0-2.0 min, 80% A to75% A; 2.0-2.5 min, 75% A to70% A; 2.5-3.0 min, 70% A to 65% A; 3.0-3.5 min, 65% A to 60% A; 3.5-6.0 min, washing with 60% A.

Waters definition accurate mass quadrupole time-of-flight (Q-TOF) XevoG2-S mass spectrometer (Waters MS Technologies, UK) was used for chemical profiling of TACS. The parameters of the MS system were as follows: capillary voltage, 2.8 kV; sample voltage, 40 V; extraction cone voltage, 4.0 V; desolvation gas rate, 700 L/h; gas temperature, 350 °C; source temperature, 100 °C; cone gas rate, 20 L/h; scan time, 0.2 s; and inter scan delay, 0.02 s. The leucine-enkephalin was used as the lockmass with a protonated mass to charge ratio of 556.2771 ([M + H]^+^ = 556.2771). The mass data was acquired in centroid mode from m/z 100 to m/z 1500.

**1.4 Identification of chemical compounds in TACS**

The structures of the chemical compounds in TACS were identified based on their retention behavior, accurate molecular weight, and MS fragment information. The structures were confirmed by the standard product database established by our research group and relevant literature (Table S1).

**Method 2. Antibiotic cocktail treatment**

The rats were randomly categorized into the following four groups (*n*=6 per group): control group, model group, Antibiotic group (Anti), and TACS+Antibiotic group. Since the 6^th^ week, Anti group and TACS+Anti group rats were orally administered with antibiotics solution containing 100 mg/kg vancomycin, 200 mg/kg streptomycin, 200 mg/kg ampicillin, and 200 mg/kg gentamicin, daily for 7 days. Concurrently, rats in the other groups were gavaged with the same volume of normal saline. After the 7th week, rats in Anti group and TACS+Anti group were provided with fresh water containing the antibiotics for 4 weeks. TACS+Anti group rats received TACS (50 mg/kg, 0.5 mL/100 g, i.g) , once daily for 4 weeks.

**Method 3. Culturing of *Lactobacillus reuteri***

The bacteria were inoculated in solid MRS medium and cultured in an incubator maintained at 37℃ for 48 h. Then, *L.reuteri* (ATCC 23272) was inoculated into MRS liquid medium and incubated at 37℃ for 48 h. The growth rate was estimated at different time points by measuring the absorbance of the medium at OD = 600 nm. The results are shown in Table S4 and Fig.S6.

**Supplementary Figures**

The fragmentation process of the compounds of TACS as follows:

1. Cheilanthifoline


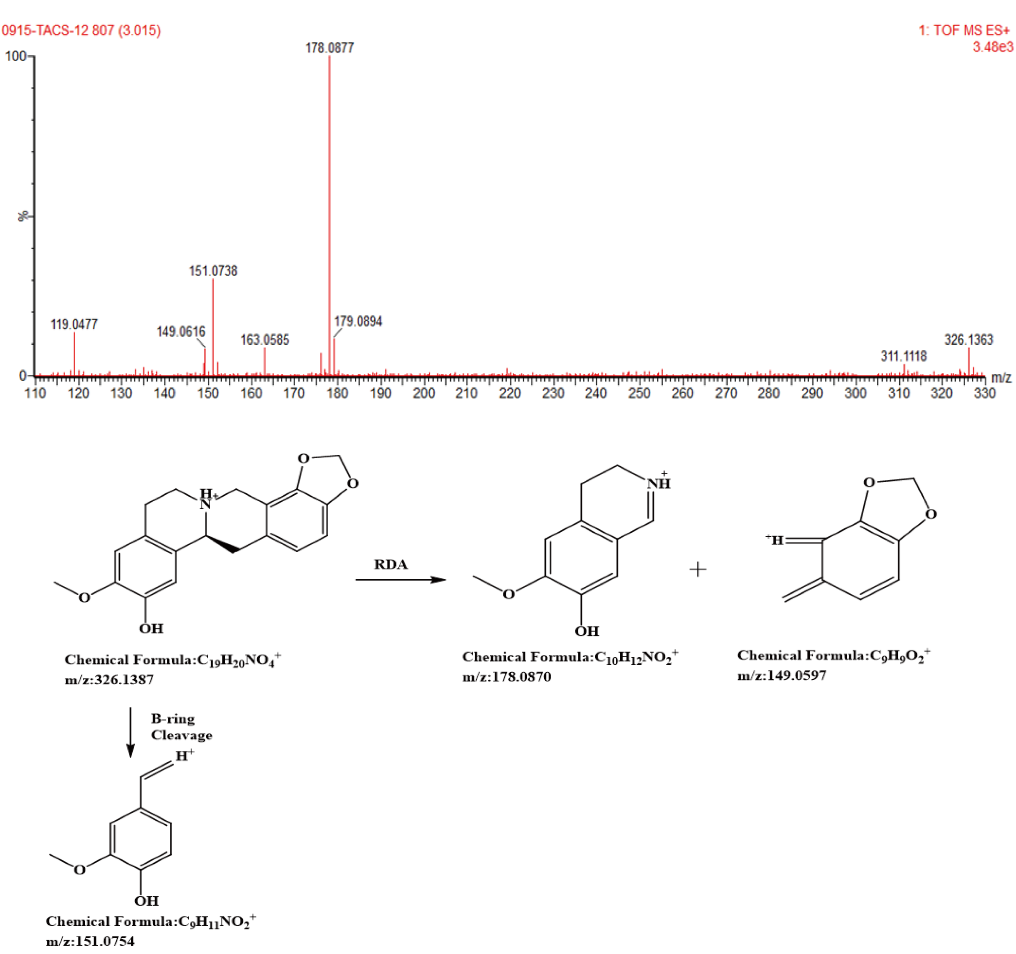


2. Berberrubine


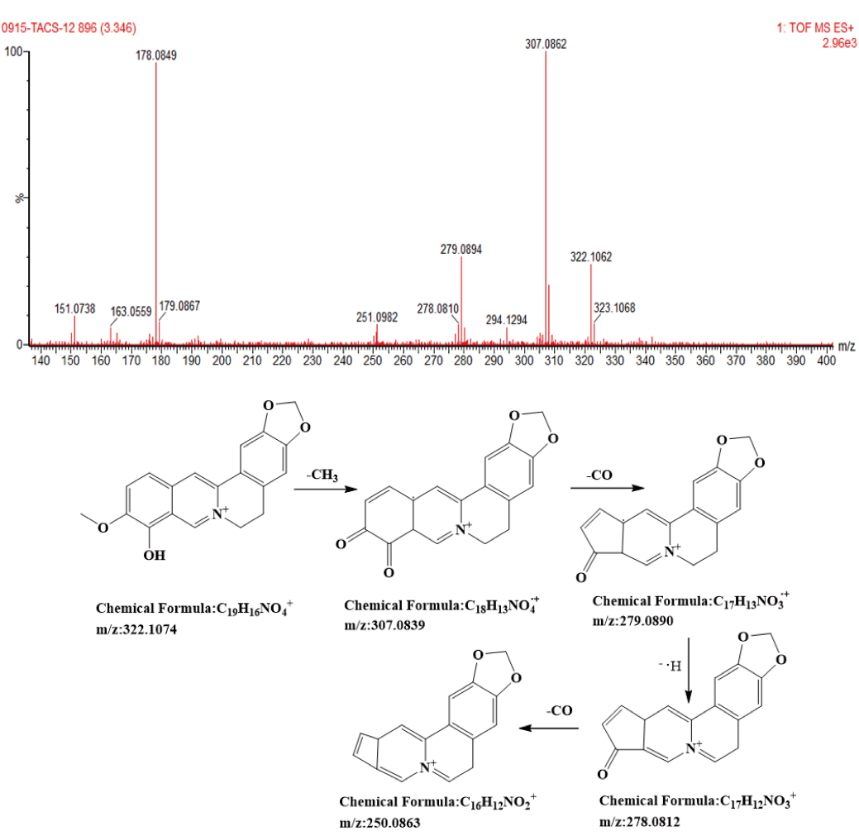


3. Epiberberine


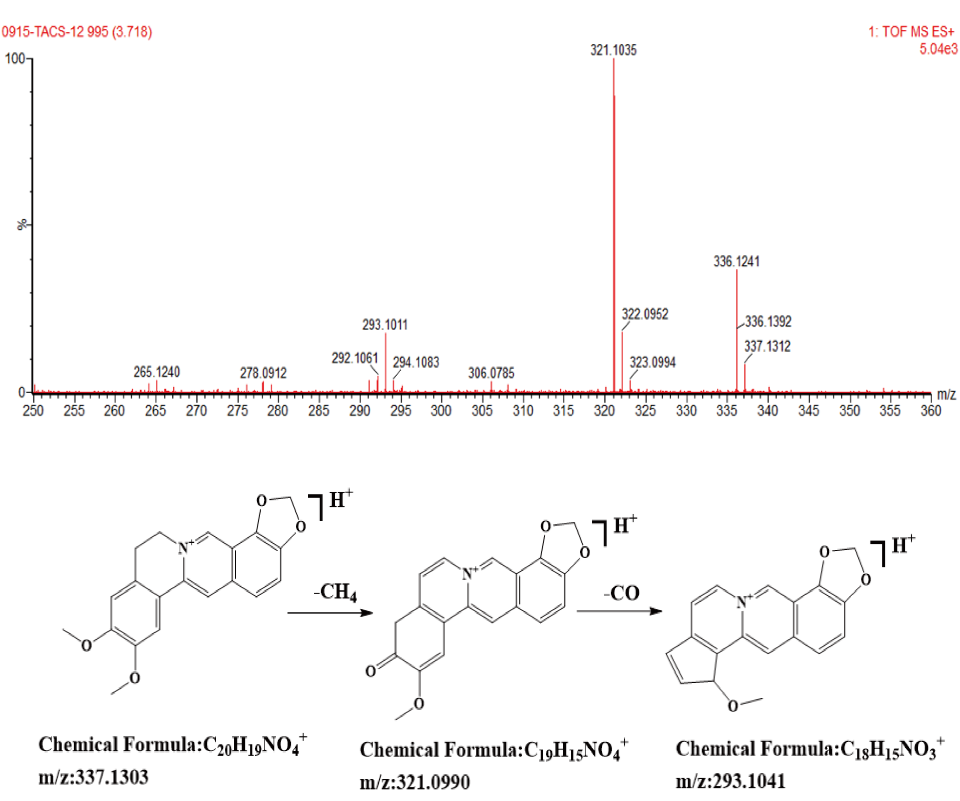


4. Tetrahydropalmatine


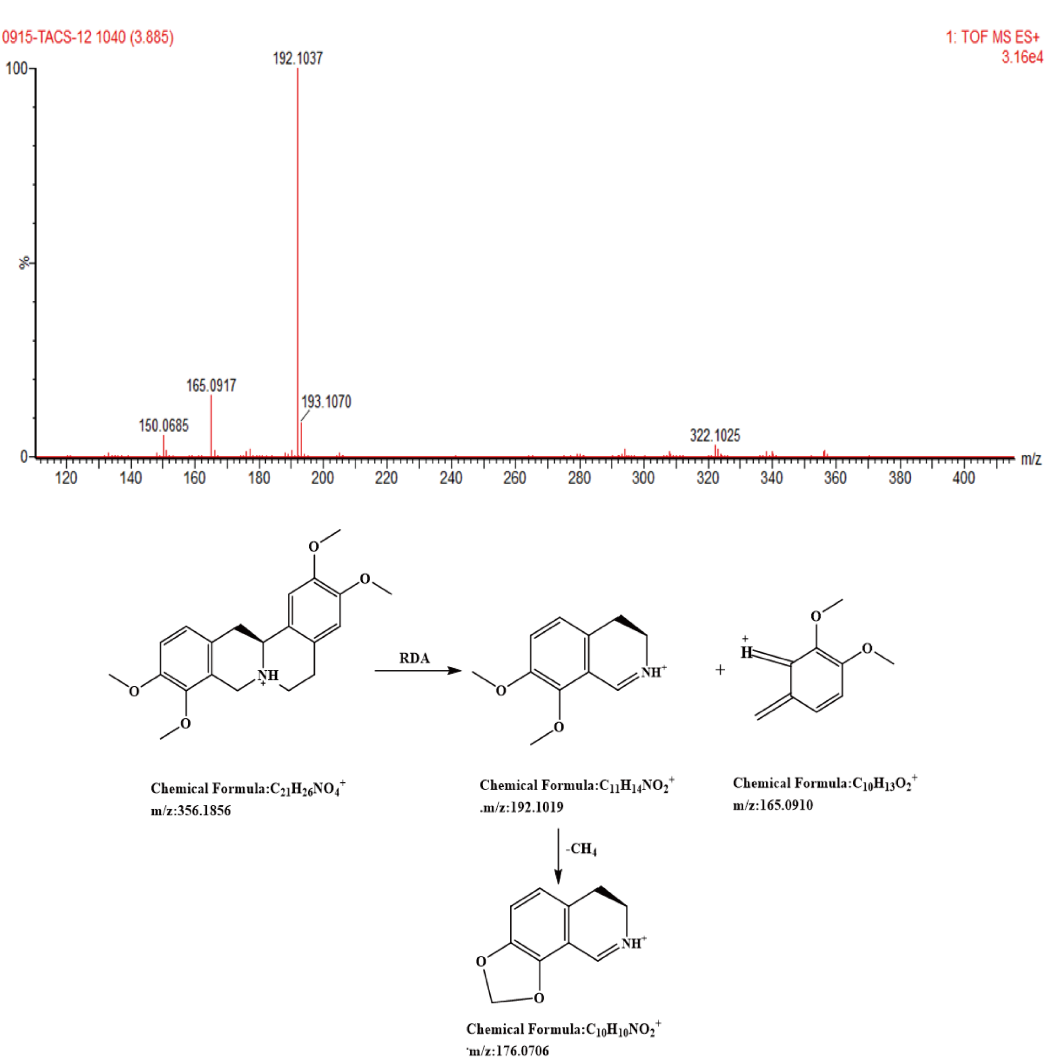


5. Jatrorrhizine


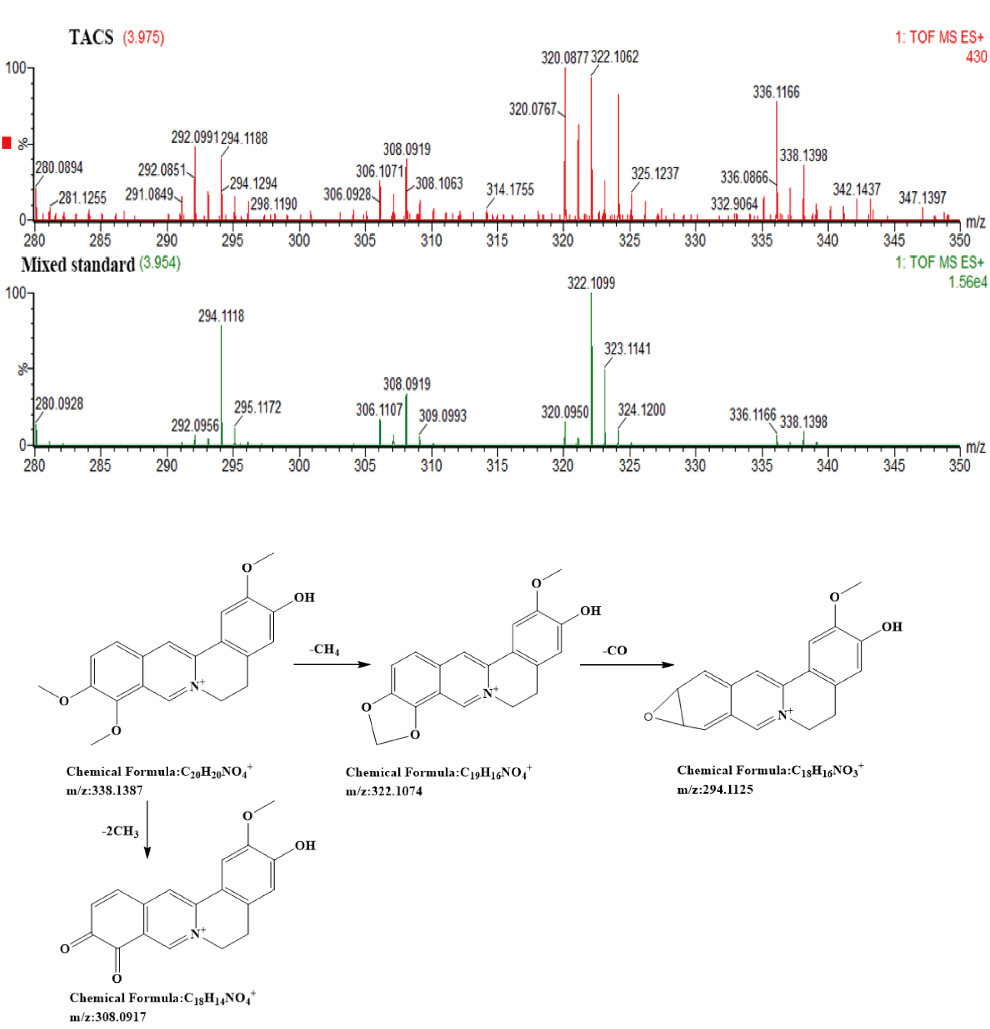


6. Coptisine


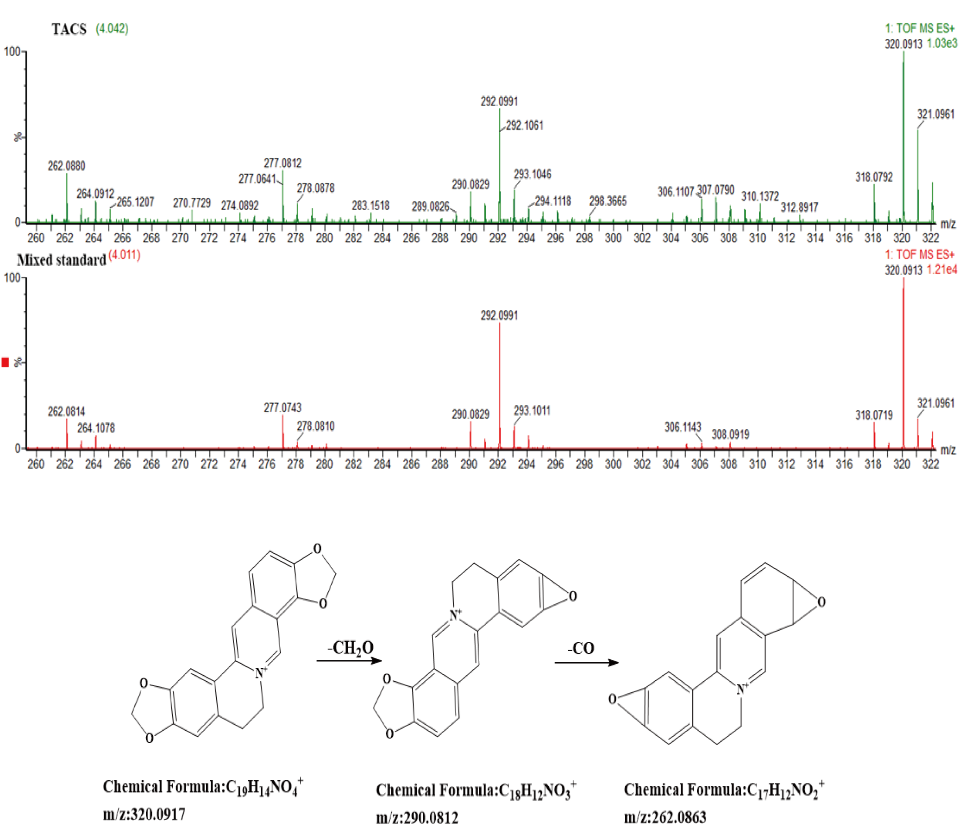


7. Dehydrocavidine


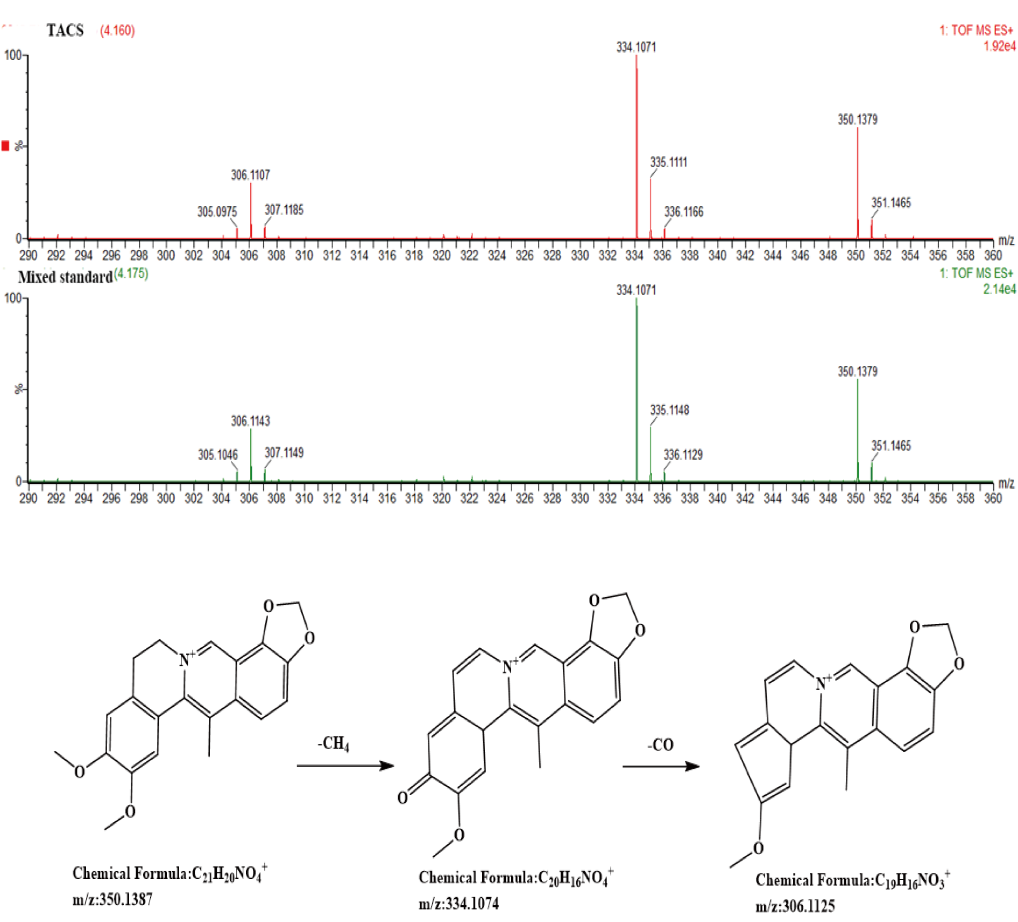


8. Palmatine


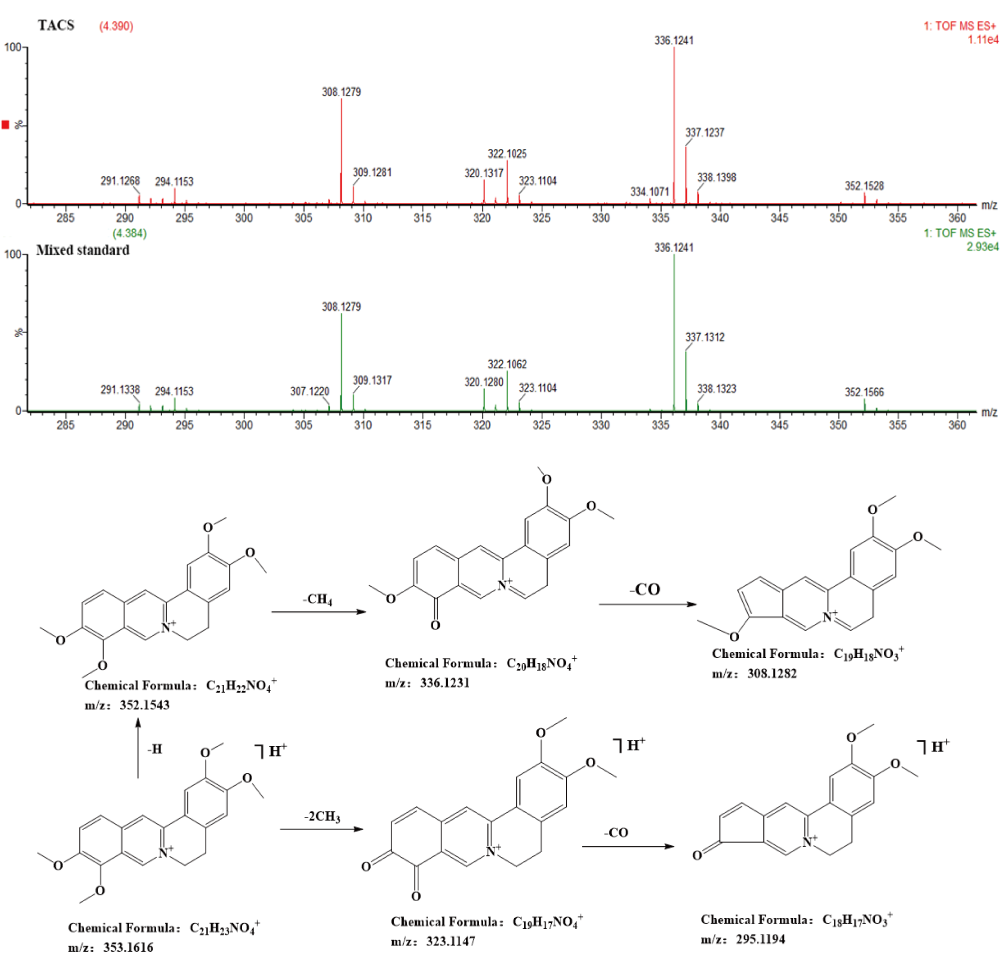


9. Berberine


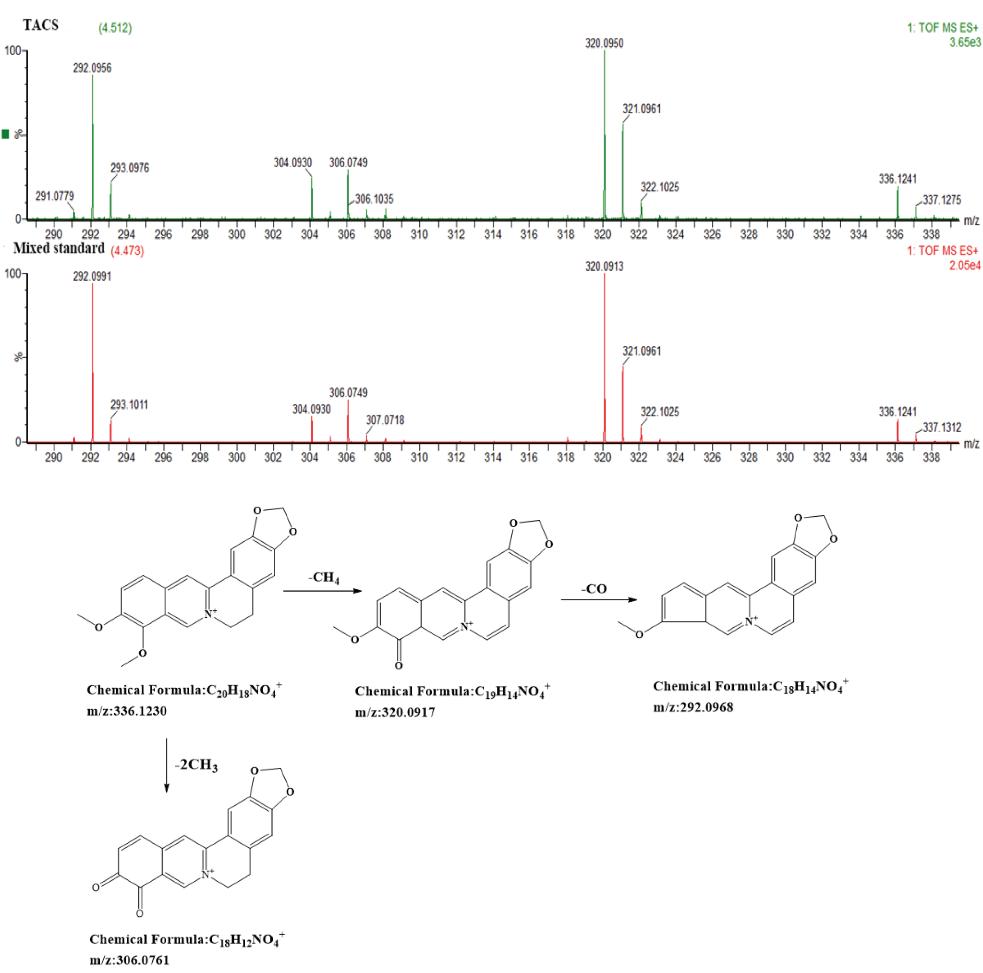


10.Chelerythrine


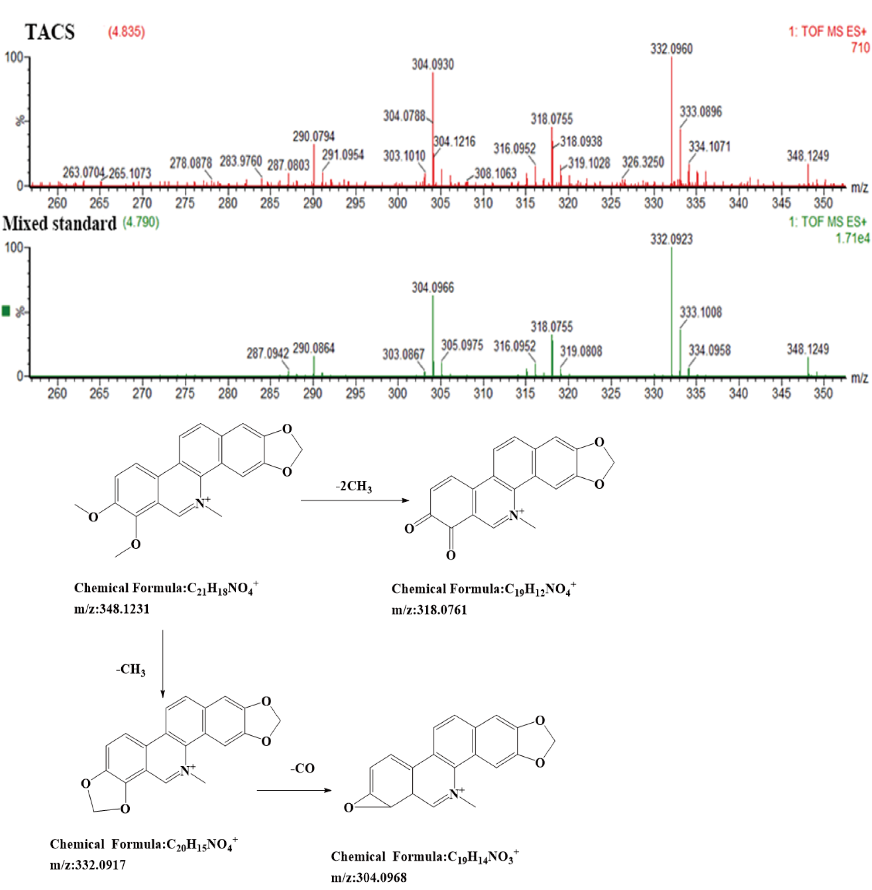


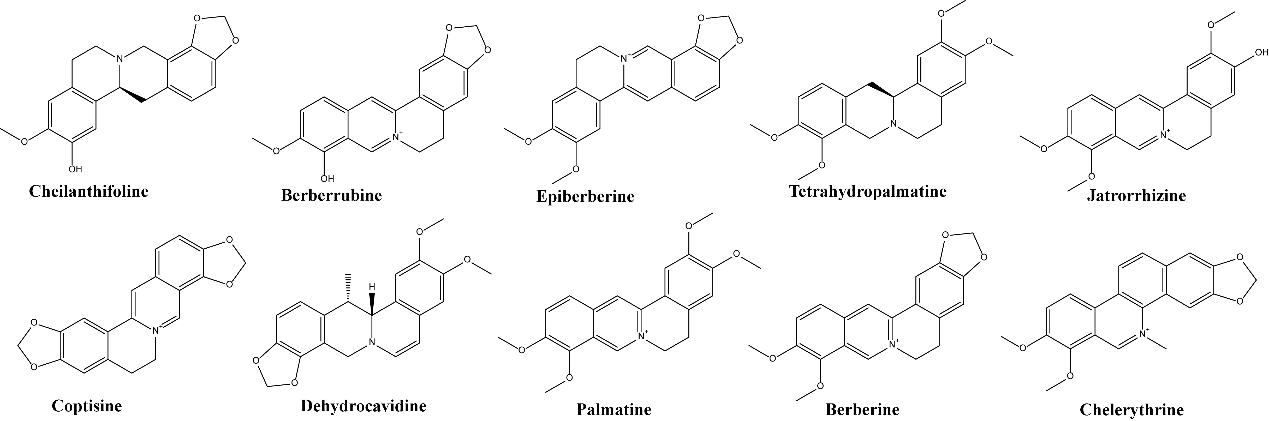


**Fig.S1.** Chemical structures of the 10 alkaloid components in TACS.


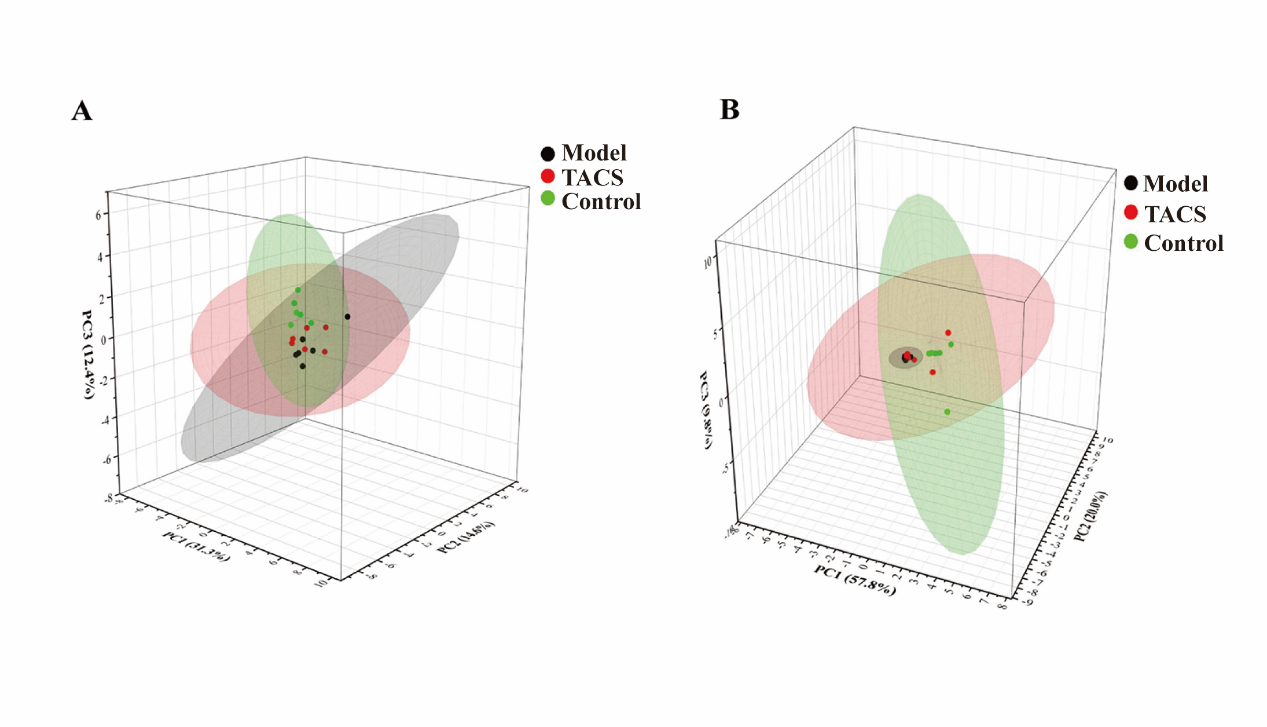


**Fig.S2** PCA analysis of liver (A) and cecal contents (B) bile acids.

**
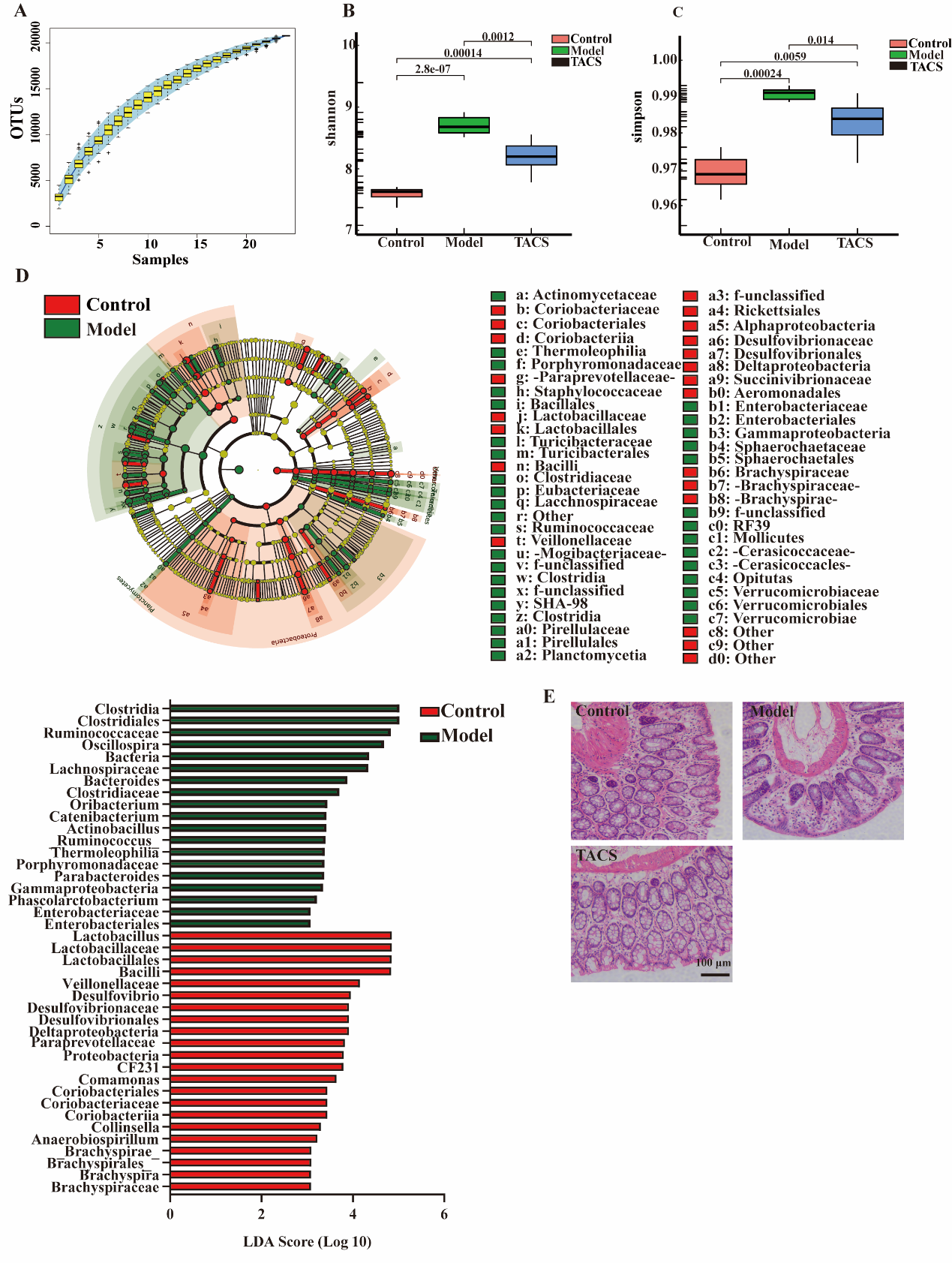
**

**Fig.S3.** TACS alleviates CCl_4_- induced gut dysbiosis. The composition of cecal microbiota in each group of rats was analyzed by 16S rRNA. (A)The specaccum accumulation curves. (B) Shannon indices(*n*=6), (C) Simpson indices(*n*=6), (D) Evolutionary branch and LEfSe analysis of the different group. (E) Representative images of colonic tissue stained with HE. (F) Occludin protein expressions was analyzed by western blotting(*n*=3). Data are shown as mean ±SEM, ^***^*P*<0.001 vs. control group, ^##^*P*<0.01vs. model group.


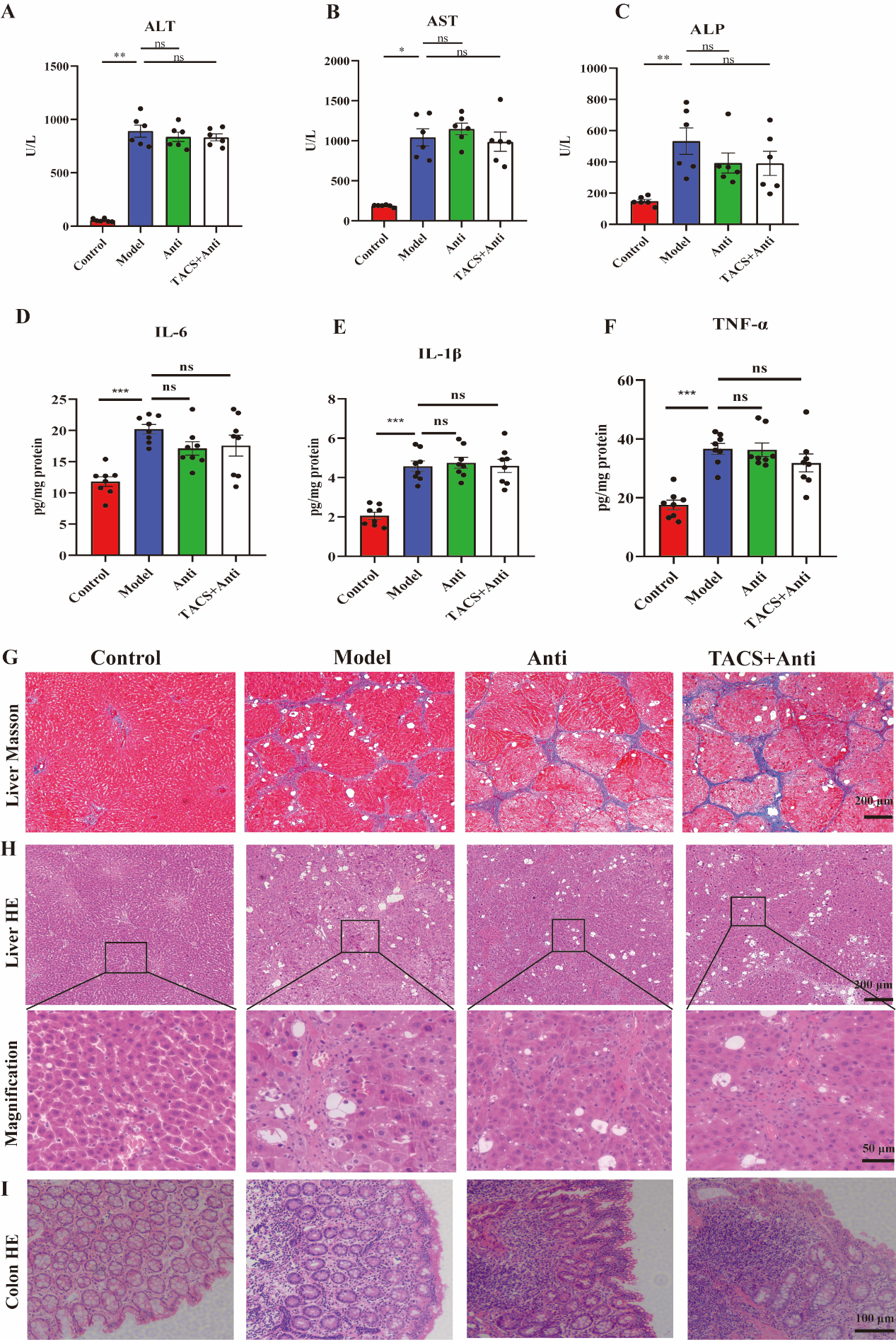


**Fig.S4.** TACS attenuates liver fibrosis in a gut microbiota–dependent manner. (A-C) Serum ALT, ALT and ALP activities (*n*=6). (D-F) The levels of IL-6, IL-1β and TNF-α in liver (*n*=8). (G,H) Representative images of liver tissues stained with Masson and HE. (I)Representative images of colonic tissue stained with HE. Data are shown as mean ±SEM, **P*<0.05, ^**^*P*<0.01, ^***^*P*<0.001 vs. control group, ns indicates no significant.


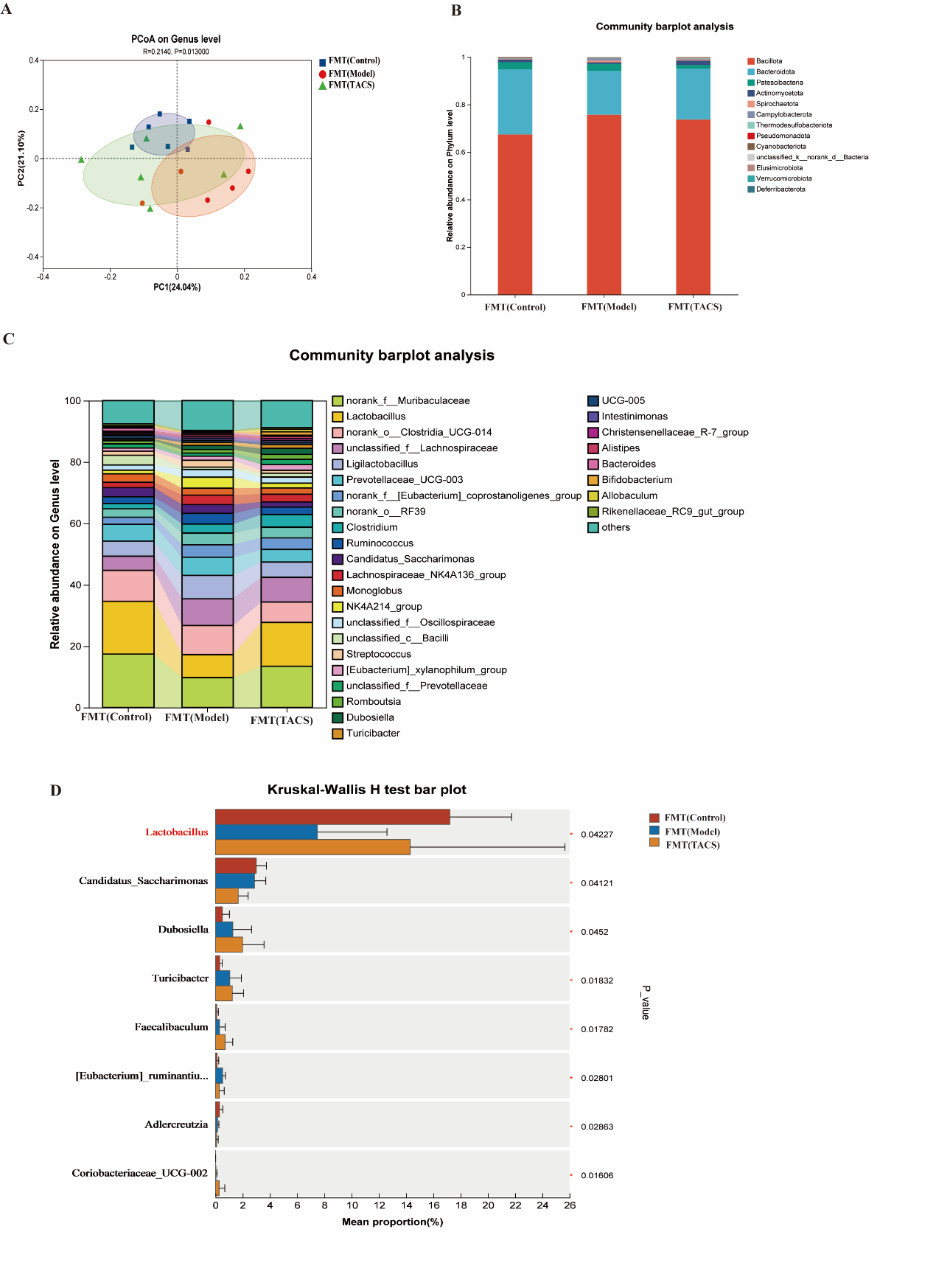


**Fig.S5.** Fecal microbiota transplantation from TACS rats regulates gut microbiota disorders in rats with liver fibrosis. (A) PCoA analysis of microbiota composition for FMT(Control), FMT(Model), and FMT(TACS) rats. (B) Bacterial profiling in the phylum level of gut microbiota from different groups. (C) Genera profiling in the phylum level of gut microbiota from different groups. (D) The top 8 abundant species with differences in abundance in FMT(Control), FMT(Model), and FMT(TACS) groups (*n*=6). Data are shown as mean ±SD, FMT(Control) *vs* FMT(Model),**P*<0.05.


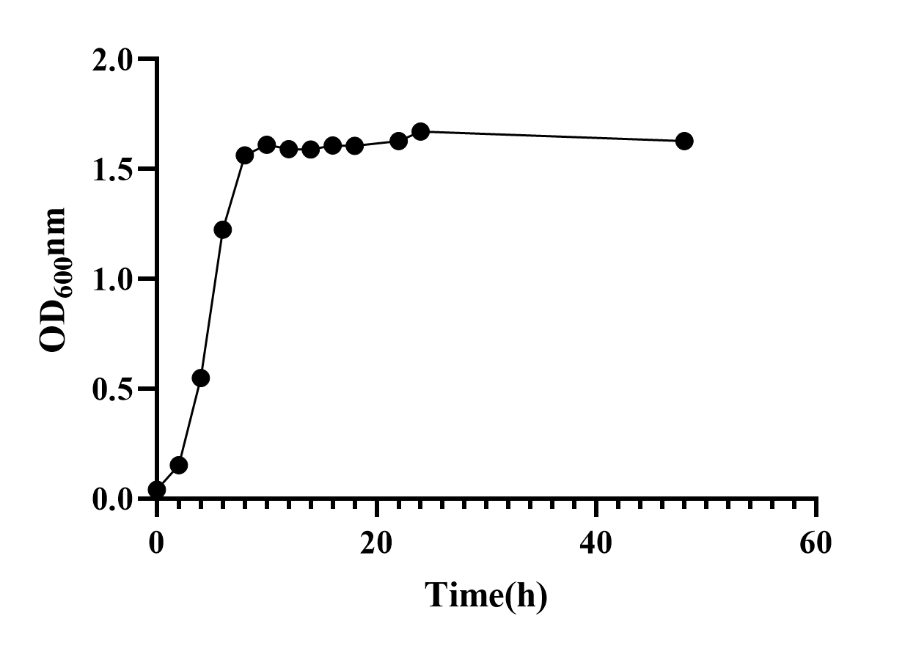


**Fig.S6** 48 h growth curve of *Lactobacillus reuteri*

**Supplementary tables**

**TableS1** Identification of the major chemical compounds in TACS (positive ion mode)

| NO | Rt/min | Compounds | Adduct ions | Formula | M.W. | Measured m/z | Calculated m/z | ppm | MS/MS data |
| --- | --- | --- | --- | --- | --- | --- | --- | --- | --- |
| 1 | 3.01 | Cheilanthifoline | [M]^+^ | C_19_H_20_NO_4_ | 326.3715 | 326.1363 | 326.1387 | 7.36 | 178.0877,149.0597,151.0754 |
| 2 | 3.34 | Berberrubine | [M]^+^ | C_19_H_16_NO_4_ | 322.1339 | 322.1062 | 322.1074 | 3.73 | 307.0862,279.0894,278.0812,250.0802 |
| 3 | 3.71 | Epiberberine | [M+H]^+^ | C_20_H_18_NO_4_ | 336.3665 | 337.1312 | 337.1303 | -2.67 | 321.1035,293.1011 |
| 4 | 3.88 | Tetrahydropalmatine | [M]^+^ | C_21_H_26_NO_4_ | 356.4415 | 356.1883 | 356.1856 | -7.58 | 192.1037,165.0910,176.0706 |
| 5 | 3.97 | Jatrorrhizine | [M]^+^ | C_20_H_20_NO_4_ | 338.3825 | 338.1398 | 338.1387 | -3.25 | 322.1062,308.0919,294.1188 |
| 6 | 4.04 | Coptisine | [M]^+^ | C_19_H_14_NO_4_ | 320.3235 | 320.0913 | 320.0917 | 1.25 | 290.0829,262.0880 |
| 7 | 4.16 | Dehydrocavidine | [M]^+^ | C_21_H_20_NO_4_ | 350.3935 | 350.1397 | 350.1387 | -2.86 | 334.1071,306.1107 |
| 8 | 4.39 | Palmatine | [M+H]^+^ | C_21_H_22_NO_4_ | 352.4095 | 353.1645 | 353.1616 | -8.21 | 352.1528,336.1241,323.1104,308.1279,295.1174 |
| 9 | 4.51 | Berberine | [M]^+^ | C_20_H_18_NO_4_ | 336.3665 | 336.1241 | 336.123 | -3.27 | 320.0950,321.0961,306.0749,292.0956 |
| 10 | 4.83 | Chelerythrine | [M]^+^ | C_21_H_18_NO_4_ | 348.3775 | 348.1249 | 348.1231 | -5.17 | 332.0960,318.0755,304.0930 |

**TableS2** The concentration of bile acids in liver of different groups of rats(*n*=6)

| Class | HMDB | KEGG | Metabolite | Control(nmol/g) | model(nmol/g) | TACS(nmol/g) |
| --- | --- | --- | --- | --- | --- | --- |
| Primary BAs | NA | NA | TαMCA | 0.8273±0.1245 | 2.4584±0.3328 | 0.9943±0.0488 |
| Primary BAs | HMDB0000932 | NA | TβMCA | 7.2291±1.0931 | 3.1900±0.6902 | 4.7337±0.4208 |
| Primary BAs | HMDB0000036 | C05122 | TCA | 4.8989±0.4066 | 11.1482±1.3764 | 12.9821±2.0991 |
| Primary BAs | HMDB0000874 | NA | TUDCA | 33.2253±4.8118 | 47.7152±5.4624 | 50.6258±7.8248 |
| Primary BAs | HMDB0000951 | C05465 | TCDCA | 0.8897±0.1029 | 0.3614±0.0598 | 0.4462±0.0455 |
| Primary BAs | HMDB0000506 | C17647 | αMCA | 0.3237±0.0822 | 0.6601±0.3272 | 0.3029±0.0802 |
| Primary BAs | HMDB0000415 | C17726 | βMCA | 0.3437±0.0732 | 1.7178±0.6799 | 0.9702±0.2665 |
| Primary BAs | HMDB0000619 | C00695 | CA | 0.6015±0.2167 | 2.3100±1.2085 | 0.8387±0.2504 |
| Primary BAs | HMDB0000138 | C01921 | GCA | 2.7286±0.4875 | 2.2331±0.6844 | 10.9279±3.7747 |
| Primary BAs | HMDB0000708 | NA | GUDCA | 0.1481±0.0409 | 0.0570±0.01940 | 0.2802±0.0850 |
| Primary BAs | HMDB0000637 | C05466 | GCDCA | 0.6408±0.1386 | 0.2350±0.0757 | 0.9212±0.2792 |
| Primary BAs | HMDB0000518 | C02528 | CDCA | 0.2054±0.0341 | 0.2423±0.0309 | 0.1617±0.0154 |
| Secondary BAs | NA | NA | TωMCA | 0.8273±0.1245 | 2.4584±0.3328 | 0.9943±0.0488 |
| Secondary BAs | NA | NA | THDCA | 5.9623±0.5533 | 18.4018±3.1548 | 6.9126±1.3721 |
| Secondary BAs | HMDB0000896 | C05463 | TDCA | 8.1811±1.3189 | 2.6656±0.4726 | 2.8888±0.3631 |
| Secondary BAs | NA | NA | TDHCA | 0.0127±0.0003 | 0.0128±0.0008 | 0.0134±0.0012 |
| Secondary BAs | HMDB0000722 | C02592 | TLCA | 0.0460±0.0145 | 0.0276±0.0067 | 0.0168±0.0037 |
| Secondary BAs | HMDB0000917 | C17644 | UCA | 0.0646±0.0316 | 0.0254±0.0121 | 0.0185±0.0026 |
| Secondary BAs | HMDB0000364 | C17727 | ωMCA | 0.0053±0.0048 | 0.1465±0.0881 | 0.0380±0.0316 |
| Secondary BAs | HMDB0000760 | NA | HCA | 0.0064±0.0012 | 0.0228±0.0052 | 0.0112±0.0019 |
| Secondary BAs | NA | NA | AlloCA | 0.0353±0.0105 | 0.0828±0.0231 | 0.0429±0.0134 |
| Secondary BAs | NA | NA | NorCA | 0.0233±0.0009 | 0.0183±0.0033 | 0.0251±0.0065 |
| Secondary BAs | NA | NA | GHDCA | 0.2729±0.0825 | 0.6172±0.2296 | 0.8875±0.0850 |
| Secondary BAs | HMDB0000631 | C05464 | GDCA | 0.1682±0.0516 | 0.1004±0.0298 | 0.3372±0.1354 |
| Secondary BAs | HMDB0000811 | C15515 | muroCA | 0.3771±0.1050 | 1.0649±0.4445 | 0.3968±0.1291 |
| Secondary BAs | HMDB0000664 | NA | bHDCA | 0.0423±0.0124 | 0.0936±0.0311 | 0.0643±0.0197 |
| Secondary BAs | NA | NA | bDCA | 0.0229±0.0021 | 0.0244±0.0031 | 0.0215±0.0023 |
| Secondary BAs | HMDB0000626 | C04483 | DCA | 0.2719±0.0138 | 0.3215±0.0175 | 0.2417±0.0143 |
| Secondary BAs | HMDB0002536 | C17661 | isoDCA | 0.0038±0.0006 | 0.0024±0.0007 | 0.0031±0.0007 |
| Secondary BAs | HMDB0000717 | C17658 | isoLCA | 0.0029±0.0001 | 0.0041±0.0007 | 0.0037±0.0005 |
| Secondary BAs | HMDB0000761 | C03990 | LCA | 0.0160±0.0024 | 0.0229±0.0022 | 0.0170±0.0028 |
| Secondary BAs | NA | NA | dehydroLCA | 0.0031±0.0005 | 0.0049±0.0005 | 0.0049±0.0013 |
| Secondary BAs | NA | NA | 6-KetoLCA | 1.1303±0.0540 | 1.1061±0.0667 | 1.0815±0.0329 |
| Secondary BAs | HMDB0000467 | NA | 7-KetoLCA | 0.0321±0.0051 | 0.0334±0.0050 | 0.0198±0.0025 |
| Secondary BAs | HMDB0000328 | NA | 12-KetoLCA | 0.0066±0.0021 | 0.0276±0.0059 | 0.0116±0.0034 |
| Secondary BAs | NA | NA | 6,7-DiketoLCA | 0.0150±0.0027 | 0.0157±0.0026 | 0.0124±0.0029 |
| Secondary BAs | NA | NA | 7,12-DiketoLCA | 0.0623±0.0051 | 0.0519±0.0048 | 0.0561±0.0057 |
| Secondary BAs | NA | NA | DHCA | 0.0050±0.0013 | 0.0039±0.0012 | 0.0029±0.0004 |
| Secondary BAs | NA | NA | 7-DHCA | 0.1153±0.0305 | 0.1952±0.1514 | 0.0584±0.0157 |
| Secondary BAs | NA | NA | 12-DHCA | 0.2294±0.1064 | 2.3337±1.1215 | 0.4783±0.2158 |
| Secondary BAs | HMDB0000502 | NA | 3-DHCA | 0.0398±0.0076 | 0.0473±0.0103 | 0.0262±0.0060 |

**TableS3** The concentration of bile acids in cecal contents of different groups of rats(*n*=6)

| Class | HMDB | KEGG | Metabolite | Control(nmol/g) | model(nmol/g) | TACS(nmol/g) |
| --- | --- | --- | --- | --- | --- | --- |
| Primary BAs | NA | NA | TαMCA | 0.1085±0.0358 | 0.0658±0.0194 | 0.3123±0.2006 |
| Primary BAs | HMDB0000932 | NA | TβMCA | 0.1244±0.0512 | 0.0472±0.0267 | 0.8651±0.6198 |
| Primary BAs | HMDB0000036 | C05122 | TCA | 0.3747±0.0891 | 0.3563±0.0999 | 1.2614±0.6551 |
| Primary BAs | HMDB0000951 | C05465 | TCDCA | 0.3628±0.0983 | 0.1175±0.0363 | 0.3365±0.0725 |
| Primary BAs | HMDB0000506 | C17647 | αMCA | 70.1179±51.2015 | 2.1255±0.5709 | 68.8767±45.0686 |
| Primary BAs | HMDB0000415 | C17726 | βMCA | 146.7296±85.6336 | 18.0370±7.64451 | 192.0693±100.4396 |
| Primary BAs | HMDB0000619 | C00695 | CA | 30.1092±25.7398 | 1.9576±0.3285 | 20.6810±11.3325 |
| Primary BAs | HMDB0000138 | C01921 | GCA | 0.8346±0.1849 | 0.0454±0.0086 | 0.7612±0.2929 |
| Primary BAs | HMDB0000637 | C05466 | GCDCA | 0.2750±0.0341 | 0.0813±0.0023 | 0.1072±0.0451 |
| Primary BAs | HMDB0000518 | C02528 | CDCA | 11.0203±7.8444 | 0.8844±0.0625 | 4.7448±2.0916 |
| Secondary BAs | NA | NA | TωMCA | 0.2205±0.1169 | 0.0506±0.0207 | 0.4711±0.3725 |
| Secondary BAs | NA | NA | THDCA | 0.5303±0.0827 | 0.2028±0.0981 | 0.8476±0.6135 |
| Secondary BAs | HMDB0000896 | C05463 | TDCA | 0.3628±0.0983 | 0.1175±0.0363 | 0.3365±0.0725 |
| Secondary BAs | HMDB0000917 | C17644 | UCA | 2.3126±1.3427 | 0.1229±0.0333 | 1.9334±1.2625 |
| Secondary BAs | HMDB0000364 | C17727 | ωMCA | 220.1666±72.2055 | 9.8632±5.2693 | 134.9148±77.0375 |
| Secondary BAs | HMDB0000760 | NA | HCA | 2.8509±1.60416 | 0.1749±0.0492 | 2.1499±1.2076 |
| Secondary BAs | NA | NA | AlloCA | 1.2432±0.9102 | 0.1805±0.0329 | 1.4681±0.9568 |
| Secondary BAs | NA | NA | NorCA | 0.2276±0.0388 | 0.0906±0.0026 | 0.2353±0.10604 |
| Secondary BAs | HMDB0000631 | C05464 | GDCA | 0.2230±0.03682 | 0.0054±0.0023 | 0.0666±0.0426 |
| Secondary BAs | HMDB0000811 | C15515 | muroCA | 214.9448±30.7644 | 20.4405±8.7102 | 70.5510±23.0214 |
| Secondary BAs | HMDB0000686 | C17662 | bUDCA | 1.1273±0.8551 | 0.1328±0.0190 | 0.8392±0.3872 |
| Secondary BAs | HMDB0000664 | NA | bHDCA | 237.1454±32.2986 | 27.4739±10.3025 | 76.5992±28.2294 |
| Secondary BAs | HMDB0000733 | NA | HDCA | 689.5635±48.8783 | 84.6418±34.1492 | 249.7871±96.1779 |
| Secondary BAs | NA | NA | bDCA | 37.4583±5.8863 | 1.0428±0.5569 | 16.5865±9.5469 |
| Secondary BAs | HMDB0000626 | C04483 | DCA | 641.5330±79.9948 | 18.5494±8.1002 | 278.0437±160.8181 |
| Secondary BAs | HMDB0002536 | C17661 | isoDCA | 0.0490±0.0205 | 0.0080±0.0040 | 0.0197±0.0071 |
| Secondary BAs | NA | NA | NorDCA | 1.0576±0.1248 | 0.0365±0.0178 | 0.2843±0.1533 |
| Secondary BAs | HMDB0000717 | C17658 | isoLCA | 52.8385±10.0658 | 1.7519±0.4968 | 8.0565±3.5102 |
| Secondary BAs | HMDB0000761 | C03990 | LCA | 234.5398±28.7004 | 6.8019±1.8185 | 48.6335±26.3561 |
| Secondary BAs | NA | NA | dehydroLCA | 54.0306±6.4569 | 6.4691±1.6990 | 16.9507±2.4748 |
| Secondary BAs | NA | NA | 6-KetoLCA | 127.8142±15.1251 | 32.5284±12.1923 | 65.4004±10.2768 |
| Secondary BAs | HMDB0000467 | NA | 7-KetoLCA | 4.6817±2.8586 | 0.3985±0.1336 | 2.5251±1.0560 |
| Secondary BAs | HMDB0000328 | NA | 12-KetoLCA | 191.6693±26.59054 | 12.9517±7.14585 | 88.8252±37.6971 |
| Secondary BAs | NA | NA | apoCA | 11.0504±1.1057 | 1.2151±0.4829 | 4.5618±1.3780 |
| Secondary BAs | NA | NA | 6,7-DiketoLCA | 0.9597±0.500 | 0.2310±0.0298 | 1.2229±0.5274 |
| Secondary BAs | NA | NA | 7,12-DiketoLCA | 2.8514±0.3710 | 0.6348±0.1145 | 2.1437±0.6707 |
| Secondary BAs | NA | NA | DHCA | 0.1396±0.0283 | 0.0493±0.0085 | 0.0856±0.0147 |
| Secondary BAs | NA | NA | 7-DHCA | 19.6554±7.8666 | 1.6395±0.4566 | 13.5337±7.1534 |
| Secondary BAs | NA | NA | 12-DHCA | 27.2718±18.6848 | 4.9684±1.0494 | 18.2363±6.0119 |
| Secondary BAs | HMDB0000502 | NA | 3-DHCA | 5.2277±3.3037 | 0.8818±0.2047 | 3.7276±3.2790 |

**TableS4** OD600 value of *Lactobacillus reuteri* under different culture time

| Time(h) | Abs-1 | Abs-2 | Abs-3 | Mean |
| --- | --- | --- | --- | --- |
| 0 | 0.0382 | 0.0419 | 0.0418 | 0.0406 |
| 2 | 0.1522 | 0.1518 | 0.1522 | 0.1521 |
| 4 | 0.5464 | 0.5493 | 0.5499 | 0.5485 |
| 6 | 1.2255 | 1.2260 | 1.2230 | 1.2248 |
| 8 | 1.5624 | 1.5637 | 1.5614 | 1.5625 |
| 10 | 1.6111 | 1.6110 | 1.6107 | 1.6109 |
| 12 | 1.5942 | 1.5899 | 1.5886 | 1.5909 |
| 14 | 1.5907 | 1.5899 | 1.5886 | 1.5897 |
| 16 | 1.6073 | 1.6075 | 1.6069 | 1.6072 |
| 18 | 1.6065 | 1.6078 | 1.6056 | 1.6066 |
| 22 | 1.6243 | 1.6274 | 1.6293 | 1.6270 |
| 24 | 1.6697 | 1.6718 | 1.6699 | 1.6705 |
| 48 | 1.6287 | 1.6283 | 1.6278 | 1.6283 |
